# Immuno-PET imaging of tumor-infiltrating lymphocytes using zirconium-89 radiolabeled anti-CD3 antibody in immune-competent mice bearing syngeneic tumors

**DOI:** 10.1371/journal.pone.0193832

**Published:** 2018-03-07

**Authors:** Denis R. Beckford Vera, Christof C. Smith, Lisa M. Bixby, Dylan M. Glatt, Stuart S. Dunn, Ryoichi Saito, William Y. Kim, Jonathan S. Serody, Benjamin G. Vincent, Matthew C. Parrott

**Affiliations:** 1 Department of Radiology and Biomedical Research Imaging Center University of North Carolina at Chapel Hill, Marsico Hall, Chapel Hill, NC, United States of America; 2 Department of Microbiology and Immunology, UNC School of Medicine, Marsico Hall, Chapel Hill, NC, United States of America; 3 Division of Hematology/Oncology, Department of Medicine, Lineberger Comprehensive Cancer Center, University of North Carolina at Chapel Hill, Marsico Hall, Chapel Hill, NC, United States of America; 4 Division of Molecular Pharmaceutics, Department of Pharmaceutical Sciences, UNC Eshelman School of Pharmacy, Marsico Hall, Chapel Hill, NC, United States of America; 5 Department of Genetics, School of Medicine, University of North Carolina at Chapel Hill, Genetic Medicine Building, Chapel Hill, NC, United States of America; 6 Department of Urology, School of Medicine, University of North Carolina at Chapel Hill, Chapel Hill, NC, United States of America; Mie University Graduate School of Medicine, JAPAN

## Abstract

The ability to non-invasively monitor tumor-infiltrating T cells in vivo could provide a powerful tool to visualize and quantify tumor immune infiltrates. For non-invasive evaluations in vivo, an anti-CD3 mAb was modified with desferrioxamine (DFO) and radiolabeled with zirconium-89 (Zr-89 or ^89^Zr). Radiolabeled ^89^Zr-DFO-anti-CD3 was tested for T cell detection using positron emission tomography (PET) in both healthy mice and mice bearing syngeneic bladder cancer BBN975. In vivo PET/CT and ex vivo biodistribution demonstrated preferential accumulation and visualization of tracer in the spleen, thymus, lymph nodes, and bone marrow. In tumor bearing mice, ^89^Zr-DFO-anti-CD3 demonstrated an 11.5-fold increase in tumor-to-blood signal compared to isotype control. Immunological profiling demonstrated no significant change to total T cell count, but observed CD4^+^ T cell depletion and CD8^+^ T cell expansion to the central and effector memory. This was very encouraging since a high CD8+ to CD4+ T cell ratio has already been associated with better patient prognosis. Ultimately, this anti-CD3 mAb allowed for in vivo imaging of homeostatic T cell distribution, and more specifically tumor-infiltrating T cells. Future applications of this radiolabeled mAb against CD3 could include prediction and monitoring of patient response to immunotherapy.

## Introduction

Successful clinical trials using blocking antibodies to the T-cell co-inhibitory receptors CTLA-4 and PD-1 have driven the recent emergence of interest in cancer immunotherapy, leading to accelerated approval timelines for several immunotherapeutic agents across multiple tumor indications [1–7]. However, the magnitude of interaction between the immune system and tumors varies greatly both within and across tumor types, resulting in differences in the response to checkpoint immunotherapy. Many of these alterations depend on the presence of impaired tumor antigen-specific effector T cells, which have been positively associated with treatment efficacy [8, 9]. Thus, one hypothesis has been that the presence of T cells within the tumor microenvironment is critical to the success of checkpoint immunotherapy.

The ability to monitor T cells within the tumor microenvironment and the immune response over the course of therapy may allow for early determination of the treatment efficacy [10, 11]. Flow cytometry, quantitative polymerase chain reaction, Vβ spectratyping, high-throughput sequencing, and immunohistochemistry are among the techniques that have provided useful information about antitumor T-cell immunity. These procedures require biopsies to evaluate the tumor immune microenvironment, greatly limiting the ability to monitor intratumoral T-cell accumulation *in vivo* or in real time. With the expanding implementation of immunotherapies, tools to monitor immune cell activity become increasingly crucial for guiding clinical decision-making and elucidating treatment options. Additionally, immune cell monitoring can be applied to chimeric antigen receptor (CAR) T cell based therapies, which have demonstrated clinical efficacy in human B cell cancers, providing a measure of both patient and donor T cell location and activity [11]. Non-invasive imaging of T cells and tumor-infiltrating lymphocytes will be an attractive means of detecting T cell infiltration and tracking the response to therapy [12]. Non-invasive monitoring could therefore change how therapies are applied and assessed, to the benefit of patients [12].

Positron emission tomography (PET) and single-photon emission computed tomography (SPECT) have been successfully used to obtain clinical images of immune cell populations [10, 13]. Other techniques such as *ex vivo* cell labeling and radiolabeled metabolic probes have also been used to non-invasively image lymphocytes. However, these approaches are not specific for T cells, have toxic effects, or simply fail to detect lymphocytes infiltrating within the tumor [14]. Immuno-PET is an emerging technique that combines the specificity of monoclonal antibodies (mAb) with the high sensitivity and quantitative potential of PET to non-invasively identify disease, stage, and response to therapy. Immuno-PET targeting of lymphocytes can provide spatial and temporal information that is currently unavailable using the standard techniques [14]. Antibodies with high affinity and specificity can be conjugated to radionuclides, and PET imaging can be used to non-invasively monitor and quantify mAb distribution in real time [15]. Zirconium-89 (^89^Zr) is a positron emitting radionuclide that has been recently approved for immuno-PET clinical studies due to its physical and biological characteristics [16]. In addition, ^89^Zr is a residualizing isotope, which prevents the isotope from leaving the target after internalization of labeled antibody [17]. Thus, ^89^Zr-immuno-PET is a powerful tool to study antigen-antibody interactions.

Recent reports have demonstrated that antibody fragments radiolabeled with ^64^Cu can non-invasively detect CD8^+^ cytotoxic T lymphocytes in mice using immuno-PET [18]. In a similar fashion, zirconium-89 radiolabeled cys-diabodies were successfully used to non-invasively detect CD4^+^ T-cell repopulation in wild-type mice and a model of immune reconstitution following hematopoietic stem cell transplantation [14]. Furthermore, ^89^Zr radiolabeled cys-diabody detected increased CD8^+^ tumor-infiltrating lymphocytes in an animal model of colon carcinoma [19]. This work demonstrated that immuno-PET targeting of CD4 and CD8 has the potential to non-invasively detect helper/regulatory and cytotoxic T-cell populations in vivo.

One limitation of the use of antibodies specific for CD4 and/or CD8 for imaging is the limited information obtained regarding the breadth of the T cell response. A more inclusive cell surface protein expressed by T cells is the pan T-cell marker CD3, which is found at all stages of T-cell development. The specificity of the CD3 antigen for T-cell lineage cells and its presence at all stages of T-cell development make CD3 a rational candidate for detecting pan-T-cell populations in vivo.

Antibodies specific for CD3 can have substantial effects on the function of CD4^+^ and CD8^+^ T cells in vivo, including induced T cell activation and expansion [20–22]. Furthermore, administration of high dose anti-CD3 mAb can preferentially deplete T cells *in vivo* [9, 22]. Therefore, while anti-CD3 mAb can be used to tag T cells, its effects *in vivo* are variable and depend on dosage, isotype, surface antigen density on target T cells, and antigen internalization/modulation of the target cell population [20–25]. Previously, zirconium-89 labeled anti-CD3 indicated a strong correlation between anti-CTLA-4–treated mice and tumor volume [26]. However, the immunological effects of radiolabeled anti-CD3 mAb at the doses used for PET imaging are still unknown. It is therefore important to elucidate the immunomodulatory effects of this novel compound to determine if it has the potential to polarize T cells toward an activated and potentially anti-tumor phenotype when used as a component of immuno-PET imaging.

We hypothesized that ^89^Zr radiolabeled anti-CD3 mAb has potential for immuno-PET detection of tumor-infiltrating T lymphocytes in mice bearing syngeneic tumors without changing overall lymphocyte numbers or viability.

## Results

### ^89^Zr-DFO-anti-CD3 reagent generation and evaluation

Full details of DFO-conjugation, radiolabeling and subsequent chemical analysis can be found in the supplementary information (S1 File). Briefly, the conjugation reaction between murine anti-CD3 mAb and DFO yielded 1.1 chelating group per protein molecule based on MALDI-TOF mass spectrometry ([m/z (DFO-anti-CD3)–m/z (anti-CD3)]/ M.W (DFO) = 148438–147606/752 = 1.1). The DFO-anti-CD3 was isolated and purified with chemical purity higher than 98%. Non-reduced SDS-PAGE for both DFO-anti-CD3 conjugate and unmodified anti-CD3 showed similar bands with apparent molecular weights of ~150kDa. Furthermore, reduced SDS-PAGE showed a modest change in apparent molecular weight between the heavy chains of the DFO-anti-CD3 conjugate and unmodified anti-CD3. This further confirmed the low degree of DFO conjugation to anti-CD3 determined by MALDI-TOF-MS. The ^89^Zr-DFO-mAb conjugates were isolated using size exclusion chromatography (SEC) with radiolabeling yields > 85% and specific activities >185 MBq/mg (>5 mCi/mg). Following SEC purification, the radiochemical purity of ^89^Zr-DFO-anti-CD3 was higher than 97%. SE-HPLC chromatograms, MALDI-TOF MS spectrums and SDS-PGE can be seen in supplementary info (S1, S2 and S3 Figs). To ensure the stability of our antibody conjugate in mouse serum, we performed an *in vitro* 72h serum stability assay. The radiochemical purity of ^89^Zr-DFO-CD3 remained higher than 98% at 72h post-incubation in C57BL/6 mouse serum. This corresponds with the lower uptake of ^89^Zr-DFO-CD3 in bone seen in the μPET/CT and ex-vivo biodistribution studies (S4 Fig). To demonstrate that labeling of antiCD3 did not alter the biological activity of the protein, a saturation binding assay was performed, resulting in a binding affinity of 89Zr-DFO-antiCD3 of 14.17 ± 3.75 nM and saturation plateau, indicating fully preserved immunoreactivity (S5 Fig).

### Biodistribution of ^89^Zr-DFO-anti-CD3, ^89^Zr-DFO-IgG2b, and ^89^Zr-DFO-IgG in healthy C57BL/6J mice

**Fig 1:**
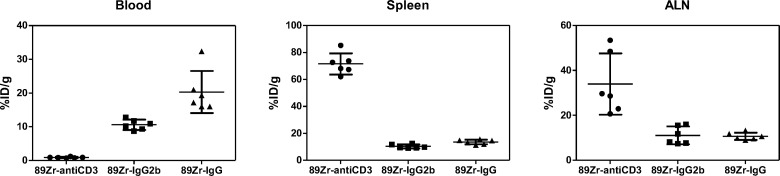
Scatter dot plots from the ex-vivo biodistribution study of ^89^Zr-DFO-anti-CD3, ^89^Zr-DFO-IgG2b, and ^89^Zr-DFO-IgG in untreated C57BL/6J mice. Each dot represents a unique mouse. Six mice (n = 6) were analyzed in each of the 3 groups for a total of 18 mice. Horizontal lines represent mean ± standard deviation. All tissue uptake data were normalized by the weight of the tissue being measured. All measurements were taken at 72 hours after injection of antibody. P-values were calculated in Table 1 using randomization permutation tests.

In order to evaluate in vivo targeting of ^89^Zr-DFO-anti-CD3, a biodistribution study was performed on 6 healthy C57BL/6J mice. Mice were intravenously injected via tail vein with 825.1 ± 14.8 kBq (22.3 ± 0.4 μCi, ~4 μg, 100 μL) of ^89^Zr-DFO-anti-CD3, or ^89^Zr-DFO-IgG2b or ^89^Zr-DFO-IgG as isotype and non-isotype specific matched heavy-chain controls, respectively. IgG2b (BE0090), an isotype control for the anti-CD3 (BE0002), was also radiolabeled with ^89^Zr and evaluated *in vivo* to ensure that the Fc region was not responsible for uptake in target organs. The biodistribution of generic ^89^Zr-IgG in C57BL/6J was likewise performed in order to determine nonspecific binding from organs involved with antibody clearance and elimination. T cells reside in the spleen, lymph nodes, thymus, and bone marrow; therefore those organs were harvested and measured for radioactivity [27]. The liver and blood were additionally harvested to monitor antibody clearance through the hepatobiliary pathway. Finally, the contrast ratio of tissue to blood was evaluated to determine in vivo imaging potential. Biodistribution results are shown in Fig 1 and Table 1. ^89^Zr-DFO-anti-CD3 showed the highest uptake in the spleen followed by the axillary lymph nodes (ALN) at 72h post-injection. Very low concentrations of ^89^Zr-DFO-anti-CD3 were measured in the blood and bone marrow at 72h post-injection.

**Table 1:** Results from the ex-vivo biodistribution of ^89^Zr-DFO-anti-CD3, ^89^Zr-DFO-IgG2b, and ^89^Zr-DFO-IgG in C57BL/6J mice (n = 6 per group).

	**% Injected dose/ gram of tissue (%ID/g)**							
	^**89**^**Zr-DFO-anti-CD3**		^**89**^**Zr-DFO-IgG2b**		^**89**^**Zr-DFO-IgG**		**Kruskal-Wallis test**	
	Mean	SD	Mean	SD	Mean	SD	**P value**	
**Blood**	0.90%	0.17%	10.61%	1.52%	20.31%	6.23%	0.0005	***
**Liver**	9.42%	0.81%	16.38%	3.71%	16.86%	4.77%	0.0062	**
**Spleen**	71.50%	7.91%	10.27%	1.47%	13.56%	1.72%	0.0009	***
**ALN**	33.96%	13.67%	11.09%	3.98%	10.68%	1.60%	0.0033	**
**Thymus**	11.99%	3.34%	7.12%	1.46%	16.16%	9.26%	0.025	*
**Bone**	0.69%	0.28%	8.95%	2.19%	0.63%	0.62%	0.0034	*
	**Tissue to blood ratios**							
	^**89**^**Zr-DFO-anti-CD3**		^**89**^**Zr-DFO-IgG2b**		^**89**^**Zr-DFO-IgG**		**Kruskal-Wallis test**	
	Mean	SD	Mean	SD	Mean	SD	**P value**	
**Liver**	10.8	2.16	1.61	0.59	0.9	0.3	0.0008	***
**Spleen**	82.59	21.59	0.97	0.08	0.7	0.13	0.0005	***
**ALN**	37.69	12.66	1.02	0.24	0.57	0.19	0.0008	***
**Thymus**	14.14	6.12	0.68	0.15	0.86	0.61	0.0033	**
**Bone**	0.76	0.31	0.86	0.27	0.03	0.03	0.0034	**

All measurements were taken at 72 hours after injection of antibody. P-values were calculated using randomization permutation tests. For pairwise comparisons

* p<0.05

** p<0.01

*** p<0.001.

To evaluate whether the uptake in target organs may be due to the interaction between ^89^Zr-DFO-anti-CD3 and T-cells, the biodistributions of both isotype control (^89^Zr-DFO-IgG2b) and IgG control (^89^Zr-DFO-IgG) were also tested in healthy C57BL/6J mice (n = 6 per group). At 72h post injection, the blood concentration of ^89^Zr-DFO-anti-CD3 was minimal (0.9% ID/g) when compared to ^89^Zr-DFO-IgG2b (10.61%) and ^89^Zr-DFO-IgG (20.31%) controls. Indicating rapid clearance and/or uptake of ^89^Zr-DFO-anti-CD3 (Fig 1, Table 1). Moreover, localization of ^89^Zr-DFO-anti-CD3 to T cell rich organ like spleen, and lymph nodes showed significantly higher accumulation than the controls. Finally, liver uptake for ^89^Zr-DFO-anti-CD3 was significantly lower than ^89^Zr-DFO-IgG2b and ^89^Zr-DFO-IgG. Whole counts of the thymus did not show large statistical differences, however, when the thymus to blood ratio was considered; the ^89^Zr-DFO-anti-CD3 was statically higher than either of the two controls. High axillary lymph node (ALN) to blood, spleen to blood and thymus to blood ratios observed for ^89^Zr-DFO-anti-CD3 confirmed high accumulation in tissue known to have high T cell counts.

### PET/CT studies of ^89^Zr-DFO-anti-CD3 in healthy C57BL/6J mice

**Fig 2:**
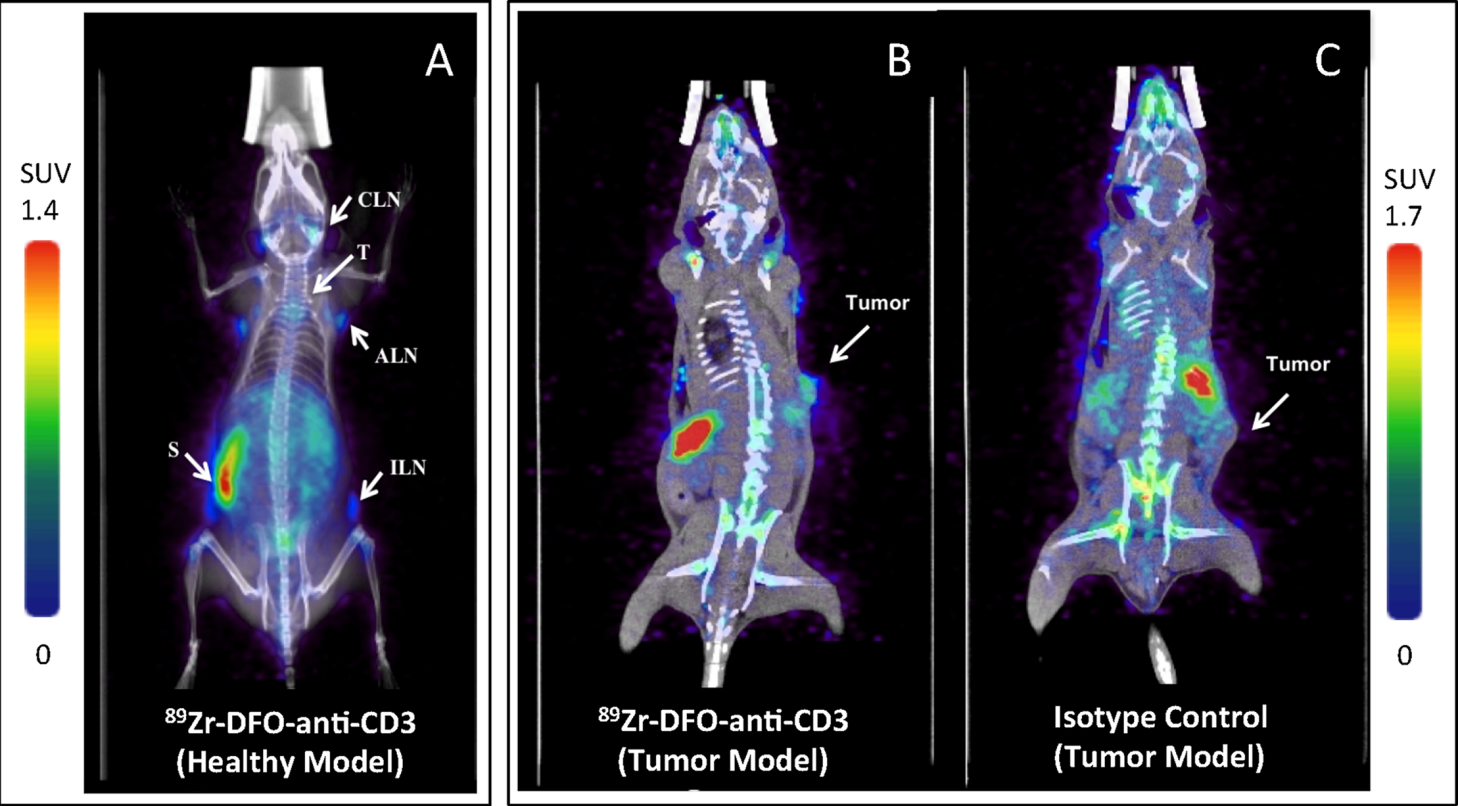
Micro-PET/CT images of ^89^Zr-DFO-anti-CD3 in C57BL/6J mice 72h post-injection (coronal view). (A) Maximum intensity projection of ^89^Zr-DFO-anti-CD3 injected into a healthy, immune-competent mouse. The radiolabeled antibody accumulated in the following lymphoid organs: cervical lymph nodes (CLN), thymus (T), axillary lymph nodes (ALN), inguinal lymph nodes (ILN) and spleen (S). (B) Micro-PET/CT (coronal slice) of ^89^Zr-DFO-anti-CD3 in C57BL/6J mice bearing BBN975 tumors and (C) a coronal slice of isotype control ^89^Zr-DFO-IgG2b in C57BL/6J mice bearing BBN975 tumors. Six mice (n = 6) in each of the 3 groups were imaged using PET/CT. SUV color bar on the left-hand side of the figure corresponds with subfigure A; SUV color bar on the right-hand side of the figure corresponds to subfigures B and C. Additional scans can be seen in the supplementary information.

Following the promising results from the biodistribution study, a microPET/CT study was performed on six healthy C57BL/6J mice (n = 6) to evaluate immuno-PET imaging potential of T cells with ^89^Zr-DFO-anti-CD3. Healthy C57BL/6J mice were intravenously injected via tail vein with 5.6 ± 0.2 MBq (153.4 ± 4.1 μCi, ~25μg, 100 μL) of ^89^Zr-DFO-anti-CD3. High-contrast images were obtained at 72h post-injection (Fig 2A), where spleen, lymph nodes, and thymus were clearly visualized with low background.

### PET/CT and ex-vivo biodistribution studies of ^89^Zr-DFO-anti-CD3 and ^89^Zr-DFO-IgG2b in C57BL/6J mice bearing syngeneic tumors

Next, we tested the ability of ^89^Zr-DFO-anti-CD3 to image tumor infiltrating T cells found in C57BL/6J mice bearing BBN975 syngeneic tumors. We compared ^89^Zr-DFO-anti-CD3 versus the isotype control ^89^Zr-DFO-IgG2b using 6 mice for each group (2 groups, n = 6). Each group of C57BL/6J mice bearing BBN975 tumors were intravenously injected with 6.0 ± 0.1 MBq (162.5 ± 1.8 μCi, ~25μg, 100 μL) of ^89^Zr-DFO-anti-CD3 or ^89^Zr-DFO-IgG2b. Immuno-PET scans of ^89^Zr-DFO-anti-CD3 showed high uptake in spleen, lymph nodes, and tumor (Fig 2B). Conversely, the isotype control (^89^Zr-DFO-IgG2b) did not show tumor uptake in microPET/CT scans (Fig 2C). The isotype control also showed higher background than that for ^89^Zr-DFO-anti-CD3 due to large amount of antibody-conjugate that remained in circulation.

**Fig 3:**
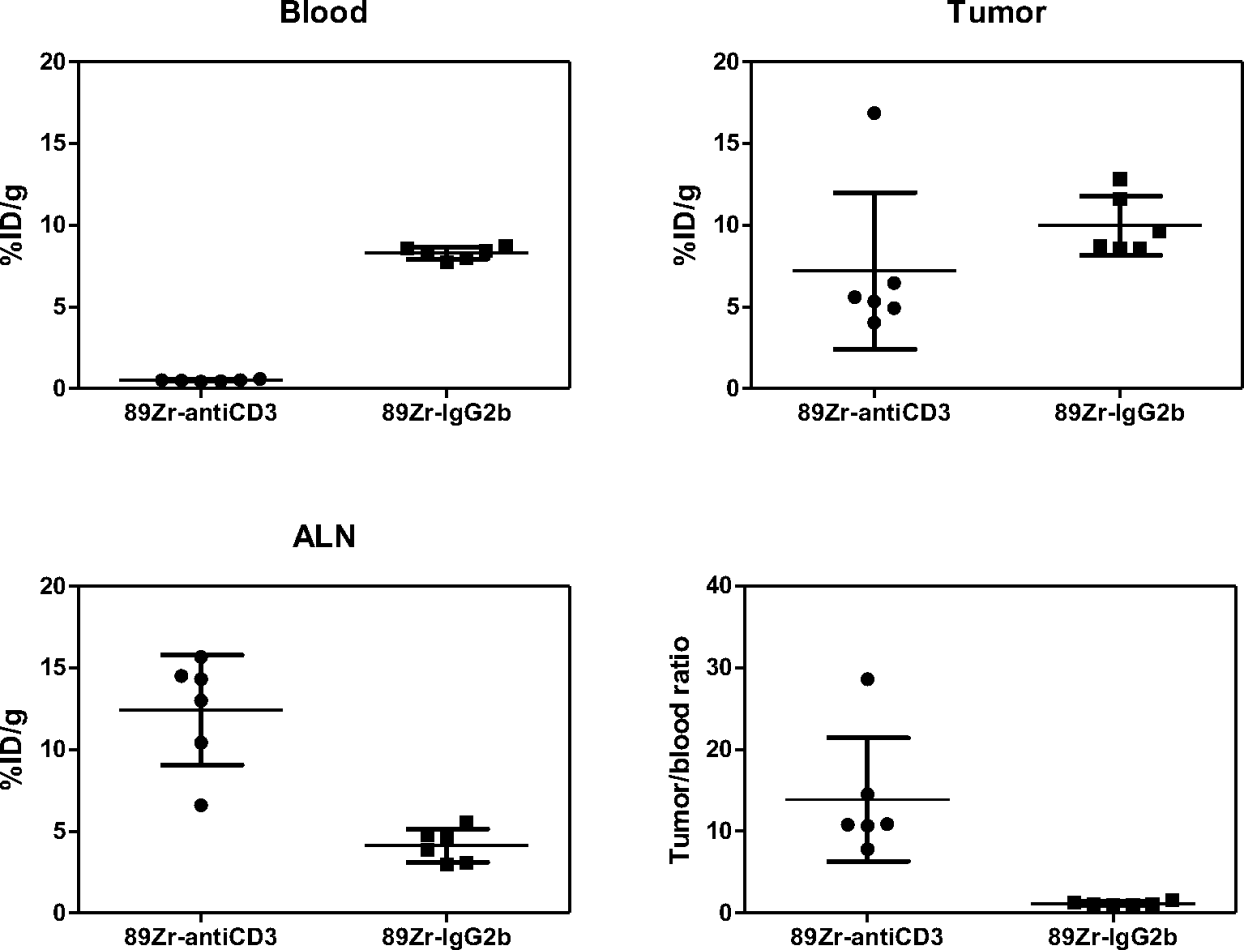
Scatter plots from the ex-vivo biodistribution study of ^89^Zr-DFO-anti-CD3 in C57BL/6J mice bearing BBN975 tumors. Each dot represents a unique mouse. Six mice (n = 6) were analyzed in each of the 2 groups for a total of 12 mice. Horizontal lines represent mean ± standard deviation. All tissue uptake data were normalized by the weight of the tissue being measured. All measurements were taken at 72 hours after injection of antibody. P-values were calculated in Table 2 using randomization permutation tests.

To further validate the microPET/CT scans, an ex-vivo biodistribution study of ^89^Zr-DFO-anti-CD3 and ^89^Zr-DFO-IgG2b in C57BL/6J mice bearing BBN975 syngeneic tumors were performed immediately following each PET scan (Fig 3). The results are summarized in Fig 3 and Table 2. Similar to the healthy-mouse study, the ^89^Zr-DFO-anti-CD3 had a significantly lower concentration in blood than the isotype control (^89^Zr-DFO-IgG2b). Also, ^89^Zr-DFO-anti-CD3 showed higher uptake in spleen, ALN, and thymus when compared against the uptake for ^89^Zr-DFO-IgG2b. Liver uptake was significantly higher for ^89^Zr-DFO-IgG2b than for ^89^Zr-DFO-anti-CD3. Although there was a not statistical difference in tumor uptake between ^89^Zr-DFO-IgG2b and ^89^Zr-DFO-anti-CD3, the tumor-to-blood ratio of ^89^Zr-DFO-anti-CD3 was 11.5-fold higher than that for ^89^Zr-DFO-IgG2b.

**Table 2:** Results from the ex-vivo biodistribution studies of ^89^Zr-DFO-anti-CD3 and ^89^Zr-DFO-IgG2b in C57BL/6J mice bearing BBN975 tumors (n = 6 per group).

	**% Injected dose/ gram of tissue (%ID/g)**							
	^**89**^**Zr-DFO-anti-CD3**		^**89**^**Zr-DFO-IgG2b**		**T-test**		**Mann Whitney Test**	
	Mean	SD	Mean	SD				
**Tumor**	7.20%	4.79%	9.99%	1.81%	2.13E-01	ns	0.0649	ns
**Blood**	0.50%	0.05%	8.31%	0.37%	2.11E-13	***	0.005	**
**Liver**	13.96%	2.45%	23.87%	3.59%	2.33E-04	***	0.0022	**
**Spleen**	51.22%	9.39%	11.98%	0.69%	1.31E-06	***	0.0022	**
**ALN**	12.42%	3.36%	4.14%	1.01%	1.77E-04	***	0.0022	**
**Thymus**	10.12%	1.70%	5.39%	0.71%	9.00E-05	***	0.0022	**
**Bone**	1.66%	0.39%	2.47%	0.86%	6.18E-02	ns	0.026	*
	**Tissue to blood ratios**							
	^**89**^**Zr-DFO-anti-CD3**		^**89**^**Zr-DFO-IgG2b**		**T-test**		**Mann Whitney Test**	
	Mean	SD	Mean	SD				
**Tumor**	13.9	7.5	1.2	0.23	2.00E-03	***	0.0022	**
**Liver**	28.1	5.98	2.88	0.49	1.23E-06	***	0.0022	**
**Spleen**	103.4	25.89	1.44	0.06	2.21E-06	***	0.0022	**
**ALN**	25.3	8.17	0.5	0.13	2.23E-05	***	0.0022	**
**Thymus**	20.49	5.11	0.65	0.07	2.50E-06	***	0.0022	**
**Bone**	3.36	0.92	0.3	0.11	1.12E-05	***	0.0022	**

All measurements were taken at 72 hours after injection of antibody. P-values were calculated using randomization permutation tests. For pairwise comparisons

* p<0.05

** p<0.01

*** p<0.001.

### Immunological effects of DFO-anti-CD3 in C57BL/6J mice

**Fig 4:**
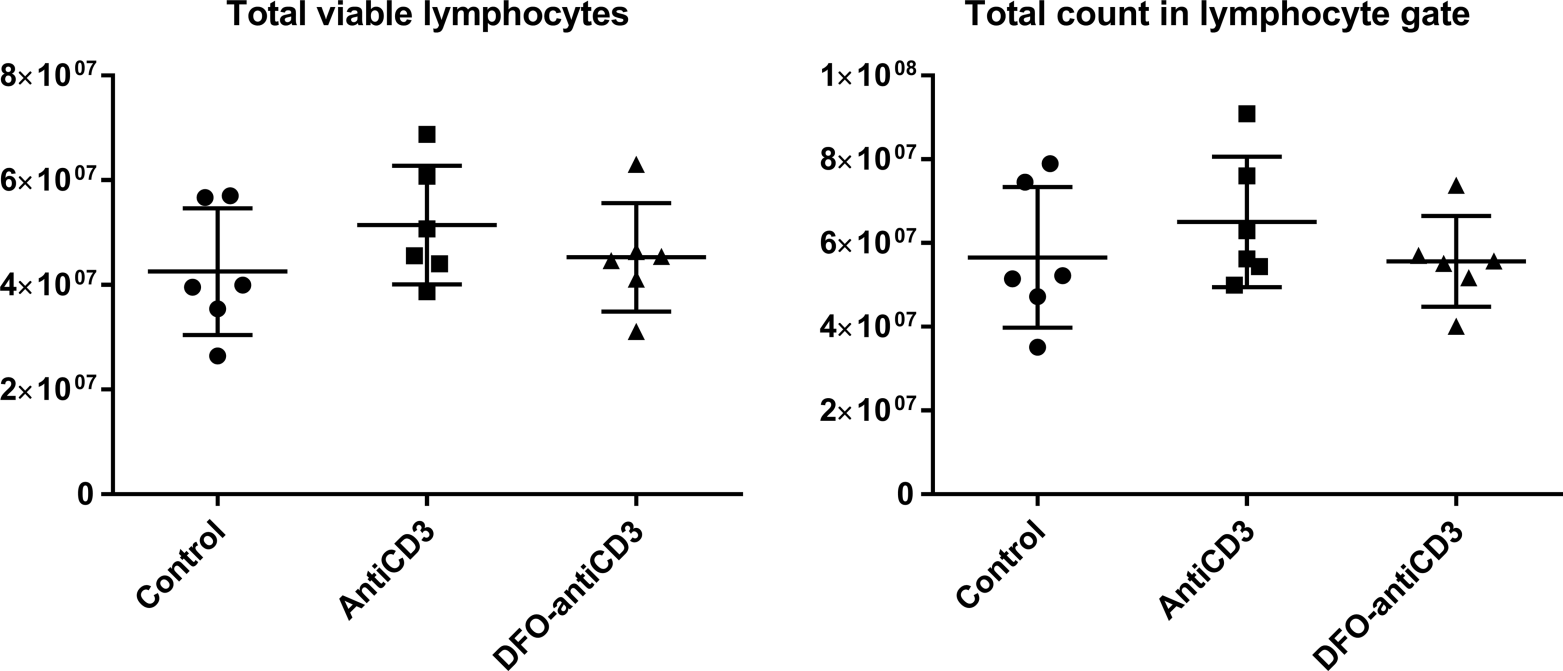
Effects of DFO-anti-CD3 and unconjugated anti-CD3 treatment on total lymphocyte numbers and viability in the spleen of C57BL/6J mice. Y-axis corresponds to absolute event counts for each respective subfigure.

To assess the immunological effects of antibodies used in the context of T-cell imaging, flow cytometric analysis of splenocytes from mice dosed with 25 μg of DFO-anti-CD3, 25 μg of native anti-CD3, or PBS control was performed. Administration of antibody showed no significant differences in lymphocyte count or viability compared to PBS control (Fig 4). These metrics demonstrate that this dose of DFO-anti-CD3 does not result in net-depletion of the splenic pan-lymphocyte population, of which T cells are a major component. To ensure free DFO did not significantly influence the T cell population within our studies, we performed a control experiment comparing T cell phenotypic subpopulation frequencies in free DFO versus PBS control treated animals. Free DFO was administered in equimolar amounts relative to DFO-anti-CD3 treated animals (0.4265 nmol), with no significant changes observed in any T cell population relative to PBS control (S7 Fig).

### DFO-anti-CD3 decreases the naïve CD8^+^ population and increases central/effector memory CD8^+^ populations

**Fig 5:**
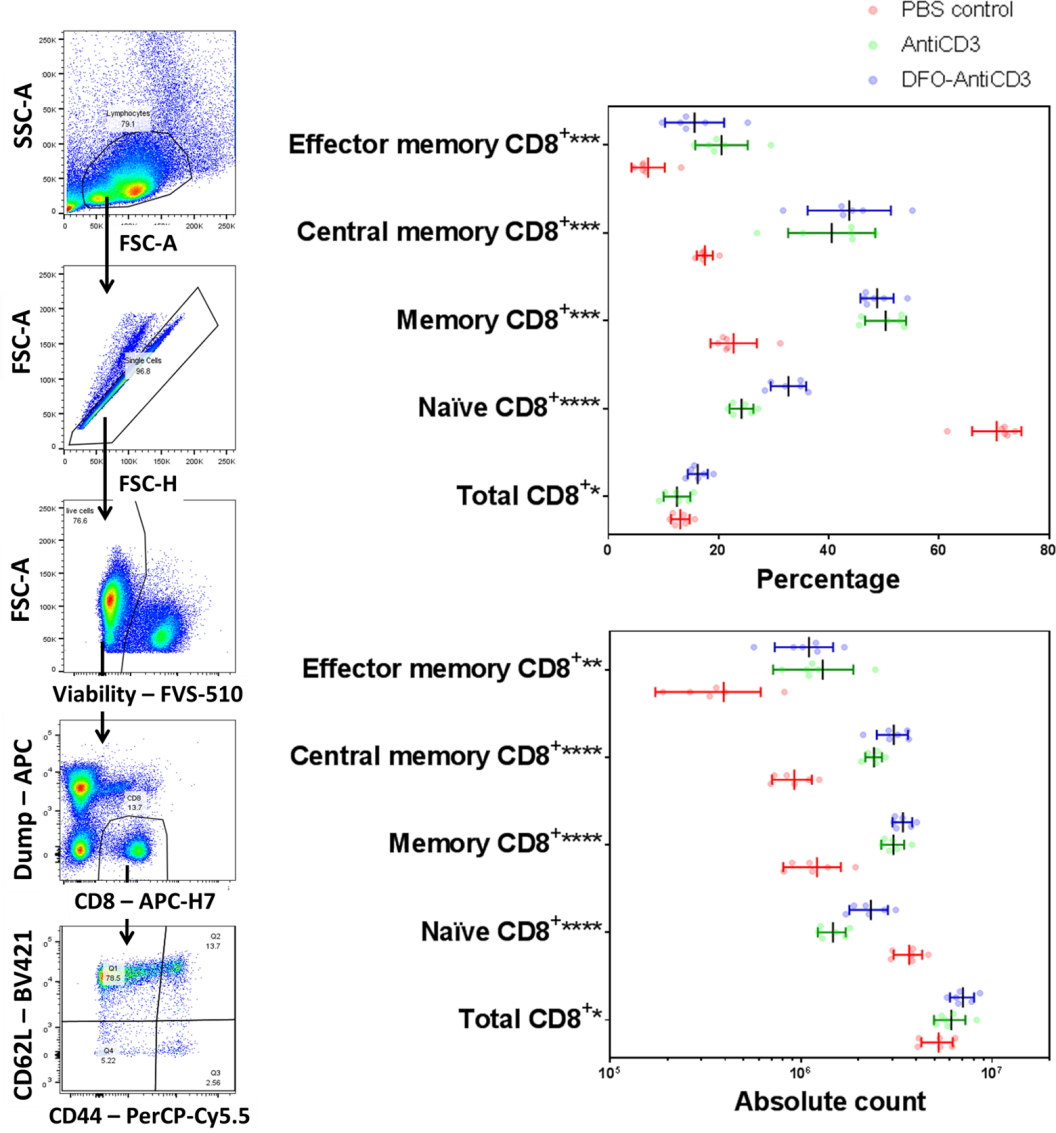
**Representative gating (left) and immunological effects (right) of DFO-anti-CD3 on CD8**^**+**^ **T-cell phenotype distribution.** Effects of DFO-anti-CD3 and unconjugated anti-CD3 treatment on frequency of total, naïve, memory, central memory, and effector memory CD8^+^ T-cells in the spleen of C57BL/6J mice are shown. Total CD8^+^ percentages and counts are with respect to all live singlets within the lymphocyte gate. Naïve, memory, central memory, and effector memory CD8^+^ percentages are with respect to total CD8^+^ populations. For all samples, statistical significance was determined via Kruskal-Wallis with a significance cutoff of * (P ≤ 0.05), ** (P ≤ 0.01), *** (P ≤ 0.001), or **** (P ≤ 0.0001), with n = 6 in all groups. Error bars represent standard deviation from the mean.

Since changes in the frequency and enumeration of peripheral lymphocytes were not observed following DFO-anti-CD3 injection, we therefore determined if the DFO-anti-CD3 altered the distribution of CD8^+^ and CD4^+^ T-cell sub-populations [28]. Specifically, naïve, total memory, central memory, and effector memory sub-populations were analyzed within the total CD8^+^ or CD4^+^ T-cell pools. The effect of DFO-anti-CD3 resulted in a modest increase in the total CD8^+^ T cell population compared to the PBS control (16.22% vs 13.07%, p < 0.05, Table 3, S1 Table). There was a small but significant increase in the percentage of CD8^+^ T cells in the DFO-anti-CD3 treated group compared to unmodified anti-CD3 treated animals (16.22% vs 12.45%, p < 0.05, Fig 5, Table 3, S1 Table).

**Table 3:** Effects of DFO-anti-CD3 and unconjugated anti-CD3 treatment on frequency of total, naïve, memory, central memory, and effector memory CD8^+^ T-cells in the spleen of C57BL/6J mice are shown.

	**By Percentage (%)**							
	**PBS Control**		**Anti-CD3**		**DFO-Anti-CD3**		**Kruskal-Wallis Test**	
	Mean	SD	Mean	SD	Mean	SD		
**Total CD8**^**+**^	13.07	1.70	12.45	2.42	16.22	1.80	0.0140	*
**Naïve CD8**^**+**^	70.48	4.48	24.17	2.18	32.68	3.22	<0.0001	****
**Memory CD8**^**+**^	22.75	4.19	50.32	3.72	48.77	3.01	0.0005	***
**Central memory CD8**^**+**^	17.50	1.45	40.53	7.90	43.72	7.56	0.0004	***
**Effector memory CD8**^**+**^	7.23	3.01	20.53	4.76	15.66	5.35	0.0002	***
	**By Absolute Count (x10**^**6**^**)**							
	**PBS Control**		**Anti-CD3**		**DFO-Anti-CD3**		**Kruskal-Wallis Test**	
	Mean	SD	Mean	SD	Mean	SD		
**Total CD8**^**+**^	5.24	0.98	6.10	1.14	7.03	1.02	0.0172	*
**Naïve CD8**^**+**^	3.68	0.63	1.47	2.45	2.32	0.53	<0.0001	****
**Memory CD8**^**+**^	1.21	0.40	3.04	4.17	3.41	0.40	<0.0001	****
**Central memory CD8**^**+**^	0.92	0.22	2.41	2.40	3.06	0.57	<0.0001	****
**Effector memory CD8**^**+**^	0.39	0.22	1.30	5.82	1.10	0.37	0.0018	**

For all samples, statistical significance was determined via Kruskal-Wallis with a significance cutoff of * (P ≤ 0.05), ** (P ≤ 0.01), *** (P ≤ 0.001), or **** (P ≤ 0.0001), with n = 6 in all groups.

When comparing population changes between the DFO-anti-CD3 treated group versus the PBS control (S1 Table), we observed a significant decrease in naïve CD8^+^ T cells both in percentage (32.68% vs 70.48%) and total count (2.32x10^6^ vs 3.68x10^6^). A corresponding increase in memory CD8^+^ T cells by percentage (48.77% vs 22.75%) and count (3.41x10^6^ vs 1.21x10^6^) was also observed. A deeper look into the central memory and the effector memory populations identified significant changes when comparing DFO-anti-CD3 treated mice against PBS treated mice. Specifically, there was a significant increase in the central memory population (43.72% vs 17.50%, p < 0.001) and a significant increase in the effector memory population (15.66% vs 7.23%, p < 0.001). All corresponding cell populations from unconjugated anti-CD3 treated animals trended similarly to the respective cell populations in DFO-anti-CD3 treated animals.

### DFO-anti-CD3 decreases total CD4^+^ T cells and may decrease naïve, central memory, and effector memory populations

**Fig 6:**
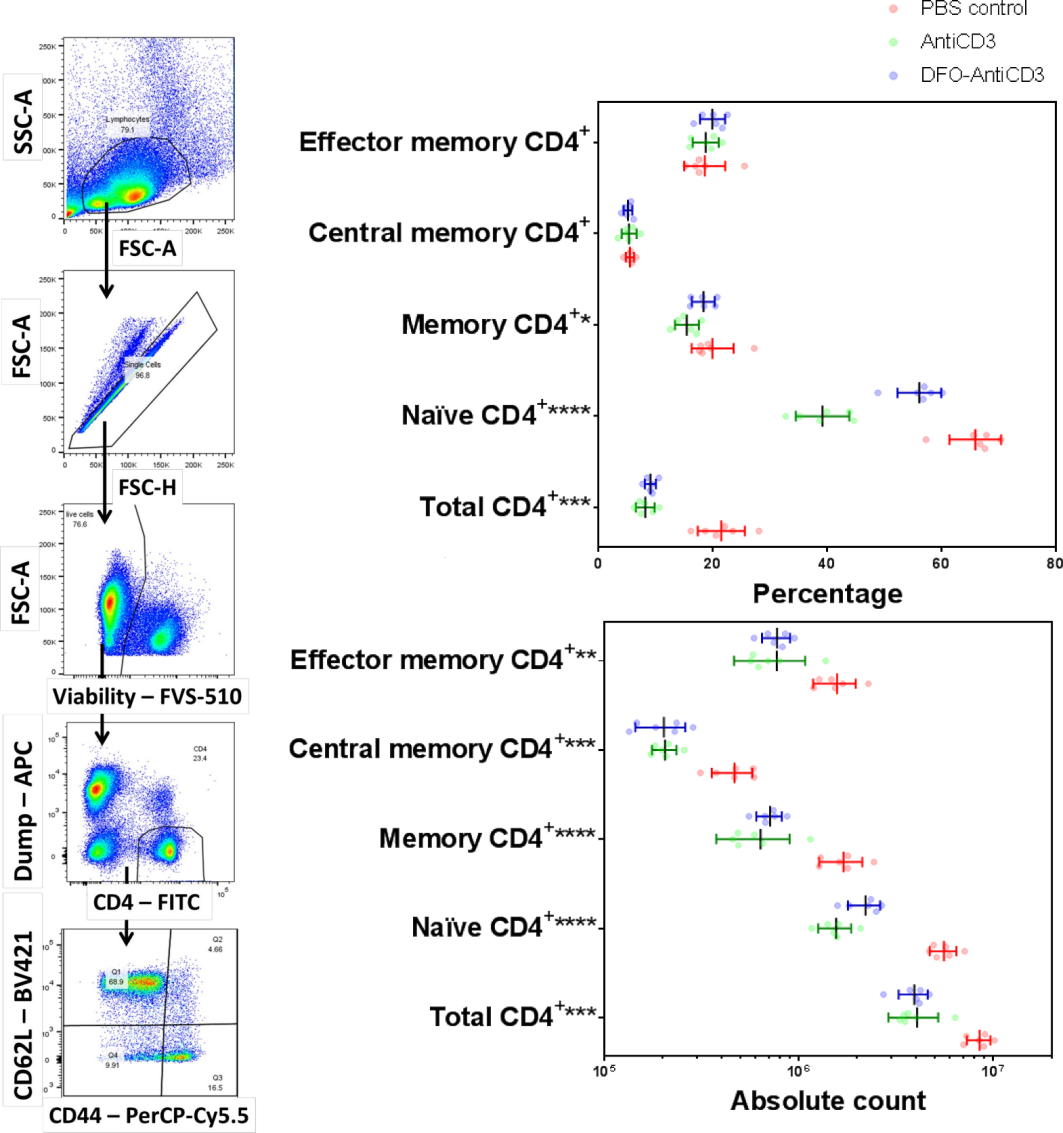
**Representative gating (left) and immunological effects (right) of DFO-anti-CD3 on CD4**^**+**^ **T-cell phenotype distribution.** Effects of DFO-anti-CD3 and unconjugated anti-CD3 treatment on frequency of total, naïve, memory, central memory, and effector memory CD4^+^ T-cells in the spleen of C57BL/6J mice. Total CD4^+^ percentages and counts are with respect to all live singlets within the lymphocyte gate. Naïve, memory, central memory, and effector memory CD4^+^ percentages are with respect to total CD4^+^ populations. For all samples, statistical significance was determined via Kruskal-Wallis with a significance cutoff of * (P ≤ 0.05), ** (P ≤ 0.01), *** (P ≤ 0.001), or **** (P ≤ 0.0001), with n = 6 in all groups. Error bars represent standard deviation from the mean.

Following interrogation of the CD8^+^ T-cell populations, we next looked at the effects of DFO-anti-CD3 on splenic CD4^+^ T cells. In contrast to the increases observed in the CD8^+^ T-cell population, total CD4^+^ T cells significantly decreased in the DFO-anti-CD3 group when compared against a PBS control (9.02% vs 21.43%, p < 0.001, Table 4, S1 Table). There were no significant differences in the percentages of naïve, total memory, central memory, or effector memory CD4^+^ T-cell sub-populations between the DFO-anti-CD3 and PBS groups (Fig 6, Table 4, S1 Table). However, all of these populations trended toward a decrease in absolute count for the DFO-anti-CD3 group likely due to the lower initial total CD4^+^ count. Changes in all CD4^+^ populations trended similarly between DFO-anti-CD3 and unconjugated anti-CD3 treated groups, without significant differences in frequency or count.

**Table 4:** Effects of DFO-anti-CD3 and unconjugated anti-CD3 treatment on frequency of total, naïve, memory, central memory, and effector memory CD4^+^ T-cells in the spleen of C57BL/6J mice.

	**By Percentage (%)**							
	**PBS Control**		**Anti-CD3**		**DFO-Anti-CD3**		**Kruskal-Wallis Test**	
	Mean	SD	Mean	SD	Mean	SD		
**Total CD4**^**+**^	21.43	4.11	8.14	1.63	9.02	0.97	0.0003	***
**Naïve CD4**^**+**^	65.83	4.52	39.13	4.68	56.05	3.86	<0.0001	****
**Memory CD4**^**+**^	19.90	3.66	15.40	2.08	18.28	2.02	0.0210	*
**Central memory CD4**^**+**^	5.46	0.74	5.31	1.31	5.11	0.78	0.8120	ns
**Effector memory CD4**^**+**^	18.53	3.59	18.70	2.28	19.88	2.20	0.4234	ns
	**By Absolute Count (x10**^**6**^**)**							
	**PBS Control**		**Anti-CD3**		**DFO-Anti-CD3**		**Kruskal-Wallis Test**	
	Mean	SD	Mean	SD	Mean	SD		
**Total CD4**^**+**^	8.48	1.17	4.05	1.16	3.92	0.67	0.0003	***
**Naïve CD4**^**+**^	5.58	0.89	1.56	0.30	2.20	0.42	<0.0001	****
**Memory CD4**^**+**^	1.70	0.43	0.64	0.26	0.71	0.11	<0.0001	****
**Central memory CD4**^**+**^	0.47	0.11	0.21	0.03	0.20	0.06	0.0006	***
**Effector memory CD4**^**+**^	1.57	0.39	0.77	0.31	0.78	0.13	0.0015	**

For all samples, statistical significance was determine via Kruskal-Wallis with a significance cutoff of * (P ≤ 0.05), ** (P ≤ 0.01), *** (P ≤ 0.001), or **** (P ≤ 0.0001), with n = 6 in all groups.

## Discussion

We report a non-invasive immuno-PET imaging study in which ^89^Zr radiolabeled anti-CD3 antibody was used to detect T-cell distributions in healthy mice and tumor-infiltrating T lymphocytes in an immune-competent animal model bearing syngeneic tumors. Our method for targeting CD3 *in vivo* provides a sensitive and specific imaging technique for detecting peripheral and tumor infiltrating T lymphocytes. To the best of our knowledge, we are the first to elucidate the immunological effects of an anti-CD3 ImmunoPET-agent on systemic T cell populations. Our results demonstrate that the CD3 antigen is a rational target to non-invasively study T cell populations *in vivo*. Further development of ^89^Zr-DFO-anti-CD3 mAb may be used to evaluate tumor-infiltrating T cells cancer patients and predict their response to therapy.

The design of an ^89^Zr-anti-CD3 immunoPET-agent required the conjugation of a bifunctional chelator to the CD3 antibody [29]. DFO was chosen as the bifunctional chelator because its complex with Zr^4+^ has high chemical and biological stability [30]. In addition, the radiolabeling of ^89^Zr with DFO-conjugates takes place at room temperature, which is convenient for protein-based conjugates [31]. The chemical and radiochemical properties of our conjugate were consistent with other ^89^Zr-DFO-mAb radioimmunoconjugates previously reported [32–34].

The ^89^Zr-DFO-anti-CD3 conjugate demonstrated a remarkable ability to target and detect tumor-infiltrating and peripheral T cells in an immune-competent syngeneic-tumor model. Absolute tumor signal was similar between ^89^Zr-DFO-anti-CD3 and the isotype control, but the ^89^Zr-DFO-anti-CD3 showed rapid clearance from the blood and yielded a tumor to blood ratio that was 11.5 times higher than the control. The combination of swift clearance and rapid protein recognition between ^89^Zr-DFO-anti-CD3 and CD3^+^ T cells resulted in the clear visualization of tumor-infiltrating and peripheral T cells by microPET/CT.

Investigation of the immunological effects of anti-CD3 and DFO-anti-CD3 on total lymphocyte frequency, number, and viability showed modest and non-significant differences compared to the PBS control. A deeper look at the immunological effects of DFO-anti-CD3 on CD4^+^ and CD8^+^ T-cell populations noted a more dramatic difference. For CD4^+^ T cells, DFO-anti-CD3 decreased the total count and percentage of the total CD4^+^ pool. This decrease was observed across all CD4^+^ sub-populations. The implication of this could be a decrease in a CD4^+^ T-cell mediated anti-tumor response, with a potentially diminished cellular (Th1/Th17) or humoral (Th2) immune function. There could also be a potential decrease in the CD4^+^ regulatory T cell population, in turn dampening the regulation of anti-tumor effector T-cell function. Further studies are needed to identify how DFO-anti-CD3 affects each of these subpopulations, and to determine whether the overall skew is in favor of an anti-tumor immune response.

Conversely, the immunological effects of DFO-anti-CD3 on CD8^+^ T-cells showed a significant increase in the total CD8^+^ pool. Our experiments suggest that DFO-anti-CD3 caused phenotypic skewing of CD8^+^ T cells from naïve into central memory (CM) and effector memory (EM) populations. Both CM and EM cells are able to recognize and target tumors for cytolysis and to yield progeny effector cells that drive a robust cytotoxic response [8]. The potential benefit of this increase in memory T cell populations is underscored by the association between tumor infiltration of memory T cells and better patient prognosis in several cancers. It is therefore possible that the systemic effects of DFO-anti-CD3 may increase activation of antigen-specific T cells, thereby increasing the number of tumor infiltrating T cell. This increase would further improve imaging capabilities and could provide secondary therapeutic effects. Further studies are needed to confirm this effect, most notably within the tumor microenvironment. Additionally, survival studies are needed to provide direct evidence that DFO-anti-CD3 treatment could have dual roles as both an imaging and therapeutic agent.

Overall, DFO-anti-CD3 demonstrated no significant change in total T cell count, but a depletion of CD4+ T cells and subsequent increase of CD8+ memory T cells. Coincidentally, a higher CD8+ to CD4+ T cell ratio has been associated with better patient prognosis in several cancers [35–37].

## Conclusions

An anti-CD3 antibody was successfully radiolabeled with ^89^Zr via DFO chelating agent. ^89^Zr-DFO-anti-CD3 was found to specifically bind to T-cells populations in healthy mice and was able to detect tumor-infiltrating T lymphocytes in C57BL/6J mice bearing syngeneic tumors. DFO-anti-CD3 showed no change in overall T lymphocyte numbers or viability, but had diminished CD4^+^ T-cell counts and polarization of the CD8^+^ T-cell pool towards a memory phenotype. These studies showed that DFO-anti-CD3 could have beneficial immunomodulatory properties favoring a more anti-tumor phenotype. Translation of this CD3-based immunoPET-agent to the clinic could provide actionable information about the tumor immune microenvironment in cancer patients, all while avoiding unwanted and invasive medical procedures.

## Materials and methods

### Synthesis and characterization of ^89^Zr-DFO-anti-CD3

An anti-CD3 antibody was modified with p–isothiocyanatobenzyl desferrioxamine (DFO) and radiolabeled with Zr-89 following a previously described method [31]. A detailed description of the synthesis, radiolabeling and characterization of ^89^Zr-DFO-anti-CD3 can be found in the supplementary information (S1 File).

### 89Zr-DFO-antiCD3 *in vitro* binding affinity assay

Immunoreactivity of 89Zr-DFO-antiCD3 was tested by binding saturation assay. C57BL/6J murine splenocytes (1.5x10^6^) in microtubes were incubated with increasing concentrations of 89Zr-DFO-antiCD3 (0.4–112 nM). Triplicate microtubes were used for each measuring point. After incubation, the suspension was centrifuged at 2000 x g for 5 min and the supernatant remove. This process was repeat 2 more times. The pellets were measured for radioactivity using an automatic γ-counter. Binding affinity (Kd) was calculated GraphPad Prism software (GraphPad Software, Inc., California, USA).

### Animal model

All animal studies were reviewed and approved by the University of North Carolina Animal Care and Use Committee (IUCAC). C57BL/6J mice (male, 4–6 weeks old, Charles River Laboratories) were used in all of the experiments. Syngeneic bladder tumors in C57BL/6J mice were induced using continuous exposure of 0.05% N-Butyl-N-(4-hydroxybutyl) nitrosamine (BBN) in drinking water [38, 39]. Tumor progression and size were monitored in the bladder by ultrasonography. Once the bladder tumors reached 5–10 mm in diameter, they were harvested and dissociated. Portions of the tumor were resuspended in growth media and plated to a 60mm plastic plate. By repeating passage, syngeneic bladder cancer cell lines, including BBN975, were successfully established and the expression of EpCAM was confirmed by flow cytometry. For immuno-PET and biodistribution assays, each C57BL/6J mouse was injected with 10 million BBN975 cells subcutaneously in the right flank. When tumors reached 50–100 mm^3^, typically 20–30 days post tumor injection, immuno-PET and biodistribution assays were performed. All animals were humanely sacrificed under CO2 asphyxiation followed by cervical dislocation, accordingly with UNC Institutional Animal Care and Use Committee (IACUC) protocol.

### Biodistribution of ^89^Zr-DFO-anti-CD3, ^89^Zr-DFO-IgG2b, and ^89^Zr-DFO-IgG in healthy C57BL/6J mice

Three groups of healthy C57BL/6J mice (n = 6) were intravenously injected via tail vein with 825.1 ± 14.8 kBq (22.3 ± 0.4 μCi, ~4 μg, 100 μL) of ^89^Zr-DFO-anti-CD3, ^89^Zr-DFO-IgG2b, or ^89^Zr-DFO-IgG. Anti-mouse CD3 mAb (BioXCell *InVivoMab*, clone 17A2) was chosen because of its availability and extensive references. Rat IgG 2b (clone BE0090) was purchased from BioXCell and Rat IgG (clone 02902) was purchased from Life technologies. Three days post-injection, we harvested blood, liver, spleen, axillary lymph node, thymus, and bone. The tissues were weighed and measured for radioactivity using a Capintec CRC-55tW dose calibrator and well counter. Whole tails were also measured for radioactivity to eliminate the variability of injections. Radioactivity measurements from tissues were decay-corrected back to the time of injection. The percent of injected dose per gram of tissue (%ID/g) were calculated using these decay-corrected radioactive counts.

### microPET/CT studies of ^89^Zr-DFO-anti-CD3 in healthy C57BL/6J mice

Healthy C57BL/6J mice were intravenously injected via tail vein with 5.6 ± 0.2 MBq (153.4 ± 4.1 μCi, ~25μg, 100 μL) of ^89^Zr-DFO-anti-CD3. At 72 hours post injection the mice were anesthetized with 2% Isoflurane/Oxygen and statically scanned by CT and PET for 30 min. CT imaging was performed at 40kV, 140uA, 360 projections per bed. MicroPET energy window 250–700 keV was used for the experiment. The CT and PET scans were co-registered using AMIDE imaging software.

### microPET/CT and ex vivo biodistribution studies of ^89^Zr-DFO-anti-CD3 and ^89^Zr-DFO-IgG2 in C57BL/6J mice bearing BBN975 tumors

Two groups of C57BL/6J mice bearing BBN975 tumors (n = 6) were intravenously injected with 6.0 ± 0.1 MBq (162.5 ± 1.8 μCi, ~25μg, 100 μL) of ^89^Zr-DFO-anti-CD3 or ^89^Zr-DFO-IgG2b. At 72h post injection, the mice were anesthetized with 2% Isoflurane/Oxygen and statically scanned by CT and PET for 30 min. After PET/CT scanning, we collected blood, liver, spleen, axillary lymph node, thymus, bone, and tumor from the treated mice. All tissues were weighed, and measured for radioactivity. The percent of injected dose per gram of tissue (%ID/g) and tumor-to-non tumor tissue (T/nT) ratios were calculated. The CT and PET scans were co-registered using AMIDE imaging software.

### Tissue dissociation

Spleens were homogenized using the GentleMACs Dissociator and the samples were passed through a 70 µM cell strainer, followed by homogenization with a 5 mL syringe plunger. The samples were centrifuged for 7 minutes at 1200 RPM, 4°C, decanting the supernatant. The remaining pellet was resuspended into 1 mL of ACK lysis buffer (150 mM NH_4_Cl, 10 mM, KHCO_3_, 0.1 nM Na_2_EDTA in DPBS, pH 7.3) for 2 minutes at room temperature before quenching with 10 mL of cold media. The samples were centrifuged for 7 minutes at 1200 RPM, 4°C, resuspended in 10 mL of cold media, and passed through a 40 μM cell strainer. Cell counting was performed by running a diluted aliquot of sample on a MACSQuant flow cytometer, counting lymphocytes as gated by forward scatter area versus side scatter area.

### Flow cytometry

Samples were washed and resuspended in cold DPBS, normalized by count, and transferred onto a 96 well V-bottom plate at 1 million lymphocytes per well. Cells were resuspended in FVS510 viability stain (1:1000 dilution in 100 μL DPBS) for 40 minutes on ice. Wells not receiving viability staining were resuspended in DPBS. Cells were washed twice in staining buffer (0.02% NaN_3_, 2% BSA in DPBS), resuspended in 50 μL Fc block (1:50 dilution in staining buffer), and incubated on ice for 15 minutes. Antibody master mix was added to samples at 50 μL per sample with final antibody concentrations of: CD3e PE (1:100; 145-2C11), CD8a APC-H7 (1:100; 53–6.7), CD4 FITC (1:200; RM4-5), CD44 PerCP-Cy5.5 (1:200; IM7), CD62L BV421 (1:200; MEL-14), NK1.1 APC (1:100; PK136), CD14 APC (1:100; rmC5-3), CD19 APC (1:100; 1D3). (All mAbs from BD Biosciences). Cells were incubated on ice for 45 minutes and washed twice with staining buffer. Cells were fixed in 2% paraformaldehyde overnight. The following morning, a minimum of 100,000 events were collected for each sample on a BD LSRFortessa flow cytometer.

### Flow cytometry analysis

FlowJo flow cytometry software Version 10 was used for analyses of all flow cytometric data (S8A Fig). Lymphocytes were identified on the 2-dimensional scatterplot of forward scatter (FSC)-area by side scatter (SSC)-area, followed by discrimination of singlet cells through FSC-area by FSC-height. Live cells were next identified by negative signal from viability staining. From this population of lymphocytes, T cells were identified as events which were CD19, CD14, and NK1.1 negative and either CD4 or CD8 positive. This strategy for T cell identification was used in place of CD3 staining due to significant decreases in CD3 median fluorescence intensity of anti-CD3 and DFO-antiCD3 treated animals (S8C and S8D Fig), presumably due to competitive binding of treatment and staining antibodies. Within CD4^+^ and CD8^+^ T cell populations, cells were identified as naïve (CD44^-^, CD62L^+^), central memory (CD44^+^, CD62L^+^), or effector memory (CD44^+^, CD62L^-^) (S9 Fig).

### Statistical analysis

Data are shown as mean ± standard deviation. Differences between multiple groups were tested for significance using Kruskal-Wallis test followed by a Bonferroni corrected Mann-Whitney U-test or Dunn’s multiple comparison post-test to compare differences between two groups (GraphPad, Prism 5). Non-parametric statistical analysis between two groups was performed using Mann-Whitney U-test (GraphPad, Prism 5). P values ≤ 0.05 were considered significant.

## Supporting information

S1 Fig **(A) MALDI-TOF MS spectrum of native anti-CD3. (B) DFO-anti-CD3 conjugate.** After ionization the MALDI-TOF MS of anti-CD3 and DFO-anti-CD3 showed the same fragmentation (peaks from right to left m/z, z = 1,2,3,4 and light chain). This demonstrated that the conjugation did not affect the integrity of the molecule. The slight differences in molecular weight between the anti-CD3 and DFO-anti-CD3 (i.e. 147,144 g/mol vs 147,721 g/mol) indicate the degree DFO modification.
(DOCX)

S2 Fig SDS-PAGE: (Std) molecular weight standards, (lane 1) native anti-CD3, (lane 2) DFO-anti-CD3 conjugate (lane 3) reduced native anti-CD3 and (lane 4) reduced DFO-anti-CD3 conjugate. The non-reduce SDS-PAGE of the anti-CD3 conjugate showed the same bands of the native anti-CD3 at the same apparent molecular weight of ~150 kDa. Reduced SDS-PAGE of the DFO-anti-CD3 conjugate also showed similar bands as the native anti-CD3 showing apparent molecular weights of ~50 and ~25 kDa. Thus confirming its purity and integrity. The slight change in molecular weight between bands 3&4 indicated modest DFO modification.
(DOCX)

S3 Fig **Size exclusion high performance liquid chromatography (SE-HPLC) of: (A) unmodified anti-CD3 (UV at 280nm), (B) DFO-anti-CD3 conjugate (UV at 280nm), (C)** ^**89**^**Zr-DFO-DFO-anti-CD3 (radioactive trace).** No significant change in antibody size was observed following chemical attachment of DFO and subsequent radiolabeling with ^89^Zr.
(DOCX)

S4 Fig iTLC chromatogram of ^89^Zr-DFO-CD3 after incubation in C57BL/6 mouse serum for (A) 1h, (B) 24h, (C) 48h, and (D) 72h.
(DOCX)

S5 Fig Binding saturation assay of 89Zr-DFO-antiCD3 demonstrating high affinity binding of 89Zr-DFO-antiCD3 to C57BL/6J murine splenocytes. Increasing concentration of 89Zr-DFO-antiCD3 were incubated with C57BL/6J murine splenocytes and 89Zr-DFO-antiCD3 specific binding was plotted against the 89Zr-DFO-antiCD3 total concentration initially incubated with C57BL/6J murine splenocytes.
(DOCX)

S6 Fig Transverse micro-PET/CT images of ^89^Zr-DFO-anti-CD3 in healthy C57BL/6J mice. PET/CT images were taken at isolated regions to highlight uptake in the spleen, axillary lymph nodes (ALN), cervical lymph nodes (CLN), and inguinal lymph nodes (ILN).
(DOCX)

S7 Fig Immunological effects of free DFO versus PBS control on T-cell phenotype distribution of total T cells and total, naïve, central memory, and effector memory CD4+ and CD8+ T-cells in the spleen of C57BL/6J mice. Total CD3+ percentages are with respect to live, singlet events within the lymphocyte gate. Total CD4+ and CD8+ percentages are with respect to total CD3+ T cells. Naïve, central memory, and effector memory percentages are with respect to total CD4+/CD8+ parent populations. For all samples, statistical significance was determined via Kruskal-Wallis with a significance cutoff of * (P ≤ 0.05), ** (P ≤ 0.01), *** (P ≤ 0.001), or **** (P ≤ 0.0001), with n = 6 in all groups. Error bars represent standard deviation from the mean.
(DOCX)

S8 Fig **Representative gating for CD4**^**+**^ **and CD8**^**+**^ **T-cell populations (A) and CD3+ populations (B).** Dump channel (B) consists of anti-NK1.1, CD14, and CD19 to gate out NK cells, APCs, and B cells, respectively. Representative (C) and total (D) CD3 median fluorescence intensities for C57BL/6J mice treated with DFO-anti-CD3, unconjugated anti-CD3, or PBS control.
(DOCX)

S9 Fig Representative gating for CD4^+^ (top) and CD8^+^ (bottom) naïve, memory, central memory, and effector memory phenotypes.
(DOCX)

S10 Fig Second example of tumor infiltrating lymphocytes imaged using micro-PET/CT. From left to right: CT, PET and PET-CT with coronal view (top) and transverse view (bottom). Radiolabeled antibody ^89^Zr-DFO-anti-CD3 was injected in C57BL/6J mice bearing BBN975 tumor and imaged 72h post-injection. T represents the location of the tumor.
(DOCX)

S11 Fig Third example of tumor infiltrating lymphocytes imaged using micro-PET/CT. From left to right: CT, PET and PET-CT with coronal view (top) and transverse view (bottom). Radiolabeled antibody ^89^Zr-DFO-anti-CD3 was injected in C57BL/6J mice bearing BBN975 tumor and imaged 72h post-injection. T represents the location of the tumor.
(DOCX)

S1 File Supporting materials and methods. Descriptions for reagents and instruments, conjugation and radiolabeling of antibodies, and *in vitro* serum stability assay.
(DOCX)

S1 Table **Statistical analysis of CD4**^**+**^ **(left) and CD8**^**+**^ **(right) T-cell phenotypes by frequency and absolute count, as determined via Kruskal-Wallis omnibus test with Dunn’s multiple comparisons post-test.** Significance is displayed as ns (P > 0.05), * (P ≤ 0.05), ** (P ≤ 0.01), or *** (P ≤ 0.001).
(DOCX)
