# Supplementary material for: Immuno-PET imaging of tumor-infiltrating lymphocytes using zirconium-89 radiolabeled anti-CD3 antibody in immune-competent mice bearing syngeneic tumors
Source: PLoS One. 2018 Mar 7;13(3):e0193832. doi: 10.1371/journal.pone.0193832 (PMC5841805; doi:10.1371/journal.pone.0193832)
Supplement: S1 Fig — (A) MALDI-TOF MS spectrum of native anti-CD3. (B) DFO-anti-CD3 conjugate. After ionization the MALDI-TOF MS of anti-CD3 and DFO-anti-CD3 showed the same fragmentation (peaks from right to left m/z, z = 1,2,3,4 and light chain). This demonstrated that the conjugation did not affect the integrity of the molecule. The slight differences in molecular weight between the anti-CD3 and DFO-anti-CD3 (i.e. 147,144 g/mol vs 147,721 g/mol) indicate the degree DFO modification. (DOCX) [file pone.0193832.s001.docx]

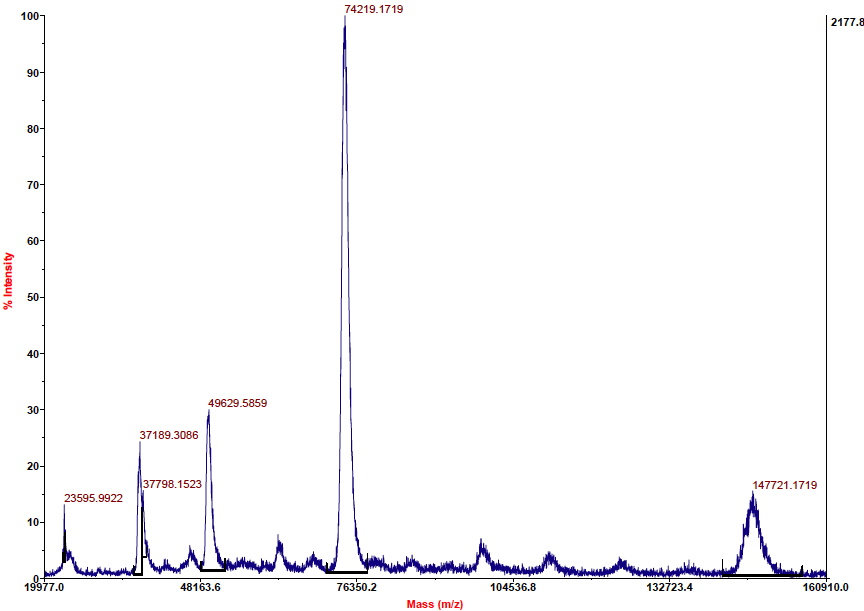

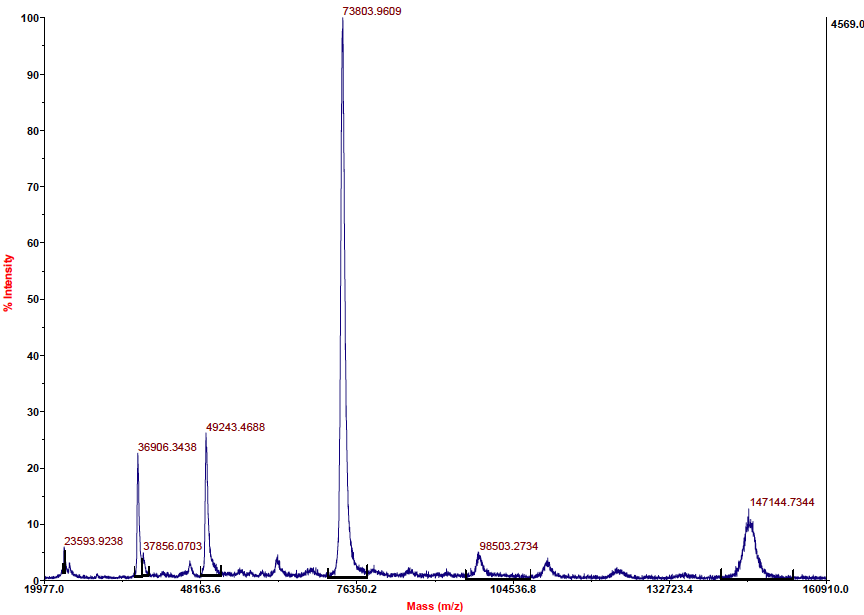


**A**

**B**

**S1 Fig:** **(A) MALDI-TOF MS spectrum of native anti-CD3. (B) DFO-anti-CD3 conjugate.** After ionization the MALDI-TOF MS of anti-CD3 and DFO-anti-CD3 showed the same fragmentation (peaks from right to left m/z, z = 1,2,3,4 and light chain). This demonstrated that the conjugation did not affect the integrity of the molecule. The slight differences in molecular weight between the anti-CD3 and DFO-anti-CD3 (i.e. 147,144 g/mol vs 147,721 g/mol) indicate the degree DFO modification.
