# Supplementary material for: Immuno-PET imaging of tumor-infiltrating lymphocytes using zirconium-89 radiolabeled anti-CD3 antibody in immune-competent mice bearing syngeneic tumors
Source: PLoS One. 2018 Mar 7;13(3):e0193832. doi: 10.1371/journal.pone.0193832 (PMC5841805; doi:10.1371/journal.pone.0193832)
Supplement: S2 Fig — The non-reduce SDS-PAGE of the anti-CD3 conjugate showed the same bands of the native anti-CD3 at the same apparent molecular weight of ~150 kDa. Reduced SDS-PAGE of the DFO-anti-CD3 conjugate also showed similar bands as the native anti-CD3 showing apparent molecular weights of ~50 and ~25 kDa. Thus confirming its purity and integrity. The slight change in molecular weight between bands 3&4 indicated modest DFO modification. (DOCX) [file pone.0193832.s002.docx]

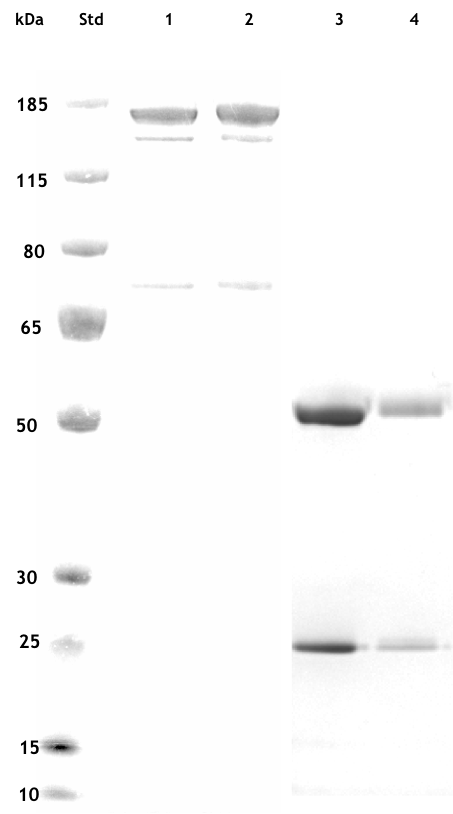


**S2 Fig:** **SDS-PAGE: (Std) molecular weight standards, (lane 1) native anti-CD3, (lane 2) DFO-anti-CD3 conjugate (lane 3) reduced native anti-CD3 and (lane 4) reduced DFO-anti-CD3 conjugate.** The non-reduce SDS-PAGE of the anti-CD3 conjugate showed the same bands of the native anti-CD3 at the same apparent molecular weight of ~150 kDa. Reduced SDS-PAGE of the DFO-anti-CD3 conjugate also showed similar bands as the native anti-CD3 showing apparent molecular weights of ~50 and ~25 kDa. Thus confirming its purity and integrity. The slight change in molecular weight between bands 3&4 indicated modest DFO modification.
