# Supplementary material for: Immuno-PET imaging of tumor-infiltrating lymphocytes using zirconium-89 radiolabeled anti-CD3 antibody in immune-competent mice bearing syngeneic tumors
Source: PLoS One. 2018 Mar 7;13(3):e0193832. doi: 10.1371/journal.pone.0193832 (PMC5841805; doi:10.1371/journal.pone.0193832)
Supplement: S3 Fig — Size exclusion high performance liquid chromatography (SE-HPLC) of: (A) unmodified anti-CD3 (UV at 280nm), (B) DFO-anti-CD3 conjugate (UV at 280nm), (C) 89Zr-DFO-DFO-anti-CD3 (radioactive trace). No significant change in antibody size was observed following chemical attachment of DFO and subsequent radiolabeling with 89Zr. (DOCX) [file pone.0193832.s003.docx]

**A**

**B**

**C**

**S3 Fig:** **Size exclusion high performance liquid chromatography (SE-HPLC) of: (A) unmodified anti-CD3 (UV at 280nm), (B) DFO-anti-CD3 conjugate (UV at 280nm), (C) ^89^Zr-DFO-DFO-anti-CD3 (radioactive trace).** No significant change in antibody size was observed following chemical attachment of DFO and subsequent radiolabelling with ^89^Zr.
