# Supplementary figures and images for: Immuno-PET imaging of tumor-infiltrating lymphocytes using zirconium-89 radiolabeled anti-CD3 antibody in immune-competent mice bearing syngeneic tumors
Source: PLoS One. 2018 Mar 7;13(3):e0193832. doi: 10.1371/journal.pone.0193832 (PMC5841805; doi:10.1371/journal.pone.0193832)

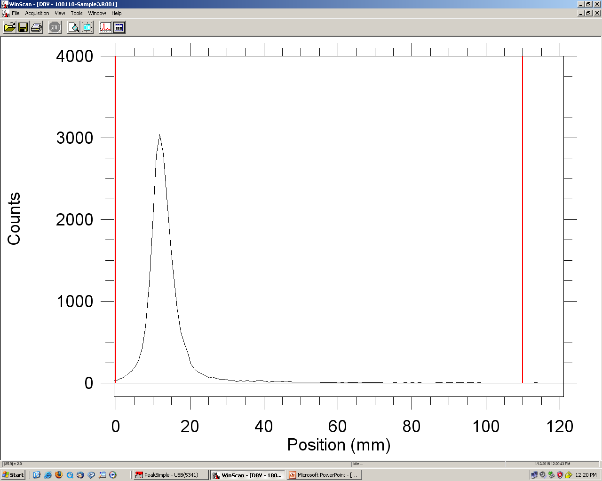

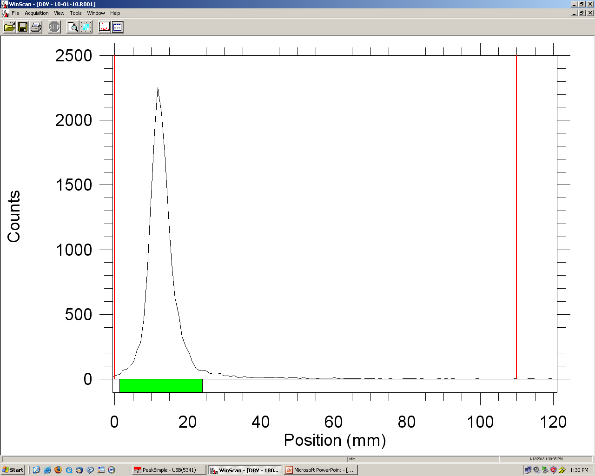

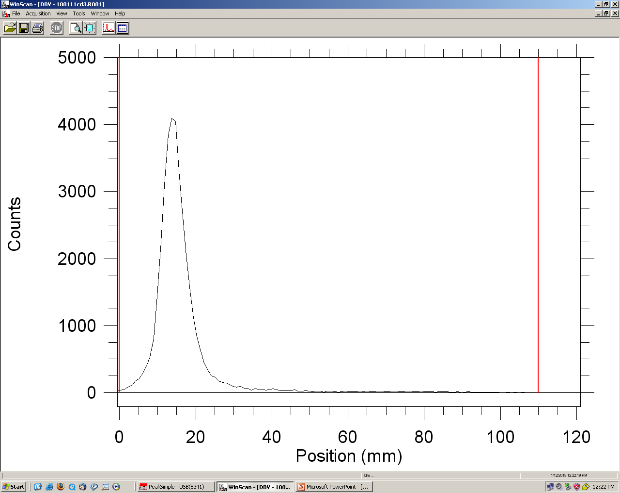

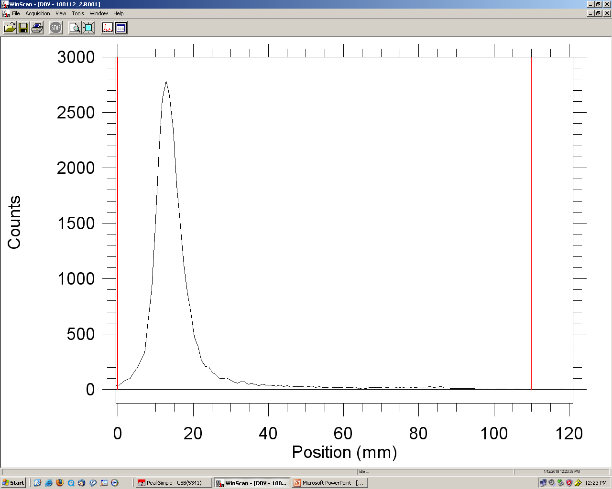


**A**

**B**

**C**

**D**

**S4 Fig**: iTLC chromatogram of 89Zr-DFO-CD3 after incubation in C57BL/6 mouse serum for (A) 1h, (B) 24h, (C) 48h, and (D) 72h.

Supplement: S4 Fig — iTLC chromatogram of 89Zr-DFO-CD3 after incubation in C57BL/6 mouse serum for (A) 1h, (B) 24h, (C) 48h, and (D) 72h. (DOCX) [file pone.0193832.s004.docx]
