# Supplementary material for: Immuno-PET imaging of tumor-infiltrating lymphocytes using zirconium-89 radiolabeled anti-CD3 antibody in immune-competent mice bearing syngeneic tumors
Source: PLoS One. 2018 Mar 7;13(3):e0193832. doi: 10.1371/journal.pone.0193832 (PMC5841805; doi:10.1371/journal.pone.0193832)
Supplement: S5 Fig — Increasing concentration of 89Zr-DFO-antiCD3 were incubated with C57BL/6J murine splenocytes and 89Zr-DFO-antiCD3 specific binding was plotted against the 89Zr-DFO-antiCD3 total concentration initially incubated with C57BL/6J murine splenocytes. (DOCX) [file pone.0193832.s005.docx]

**S5 Fig: Binding saturation assay of 89Zr-DFO-antiCD3 demonstrating high affinity binding of 89Zr-DFO-antiCD3 to C57BL/6J murine splenocytes.** Increasing concentration of 89Zr-DFO-antiCD3 were incubated with C57BL/6J murine splenocytes and 89Zr-DFO-antiCD3 specific binding was plotted against the 89Zr-DFO-antiCD3 total concentration initially incubated with C57BL/6J murine splenocytes.
