# Supplementary material for: Immuno-PET imaging of tumor-infiltrating lymphocytes using zirconium-89 radiolabeled anti-CD3 antibody in immune-competent mice bearing syngeneic tumors
Source: PLoS One. 2018 Mar 7;13(3):e0193832. doi: 10.1371/journal.pone.0193832 (PMC5841805; doi:10.1371/journal.pone.0193832)
Supplement: S6 Fig — PET/CT images were taken at isolated regions to highlight uptake in the spleen, axillary lymph nodes (ALN), cervical lymph nodes (CLN), and inguinal lymph nodes (ILN). (DOCX) [file pone.0193832.s006.docx]

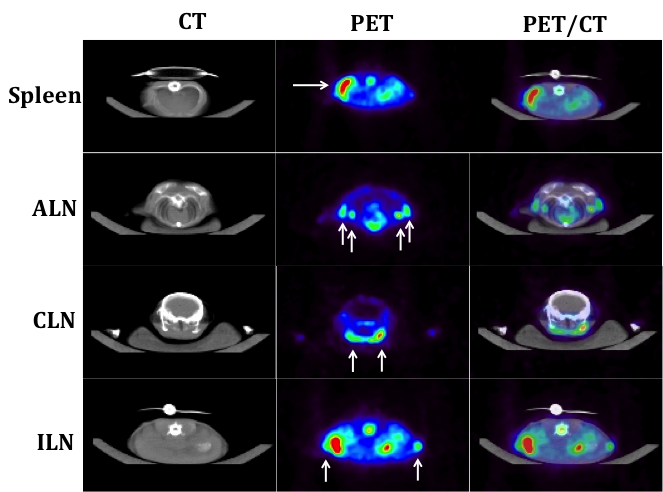


**S6 Fig**: **Transverse micro-PET/CT images of ^89^Zr-DFO-anti-CD3 in healthy C57BL/6J mice.** PET/CT images were taken at isolated regions to highlight uptake in the spleen, axillary lymph nodes (ALN), cervical lymph nodes (CLN), and inguinal lymph nodes (ILN).
