# Supplementary material for: Immuno-PET imaging of tumor-infiltrating lymphocytes using zirconium-89 radiolabeled anti-CD3 antibody in immune-competent mice bearing syngeneic tumors
Source: PLoS One. 2018 Mar 7;13(3):e0193832. doi: 10.1371/journal.pone.0193832 (PMC5841805; doi:10.1371/journal.pone.0193832)
Supplement: S7 Fig — Total CD3+ percentages are with respect to live, singlet events within the lymphocyte gate. Total CD4+ and CD8+ percentages are with respect to total CD3+ T cells. Naïve, central memory, and effector memory percentages are with respect to total CD4+/CD8+ parent populations. For all samples, statistical significance was determined via Kruskal-Wallis with a significance cutoff of * (P ≤ 0.05), ** (P ≤ 0.01), *** (P ≤ 0.001), or **** (P ≤ 0.0001), with n = 6 in all groups. Error bars represent standard deviation from the mean. (DOCX) [file pone.0193832.s007.docx]

**S7 Fig: Immunological effects of free DFO versus PBS control on T-cell phenotype distribution of total T cells and total, naïve, central memory, and effector memory CD4+ and CD8+ T-cells in the spleen of C57BL/6J mice.** Total CD3+ percentages are with respect to live, singlet events within the lymphocyte gate. Total CD4+ and CD8+ percentages are with respect to total CD3+ T cells. Naïve, central memory, and effector memory percentages are with respect to total CD4+/CD8+ parent populations. For all samples, statistical significance was determined via Kruskal-Wallis with a significance cutoff of * (P ≤ 0.05), ** (P ≤ 0.01), *** (P ≤ 0.001), or **** (P ≤ 0.0001), with n=6 in all groups. Error bars represent standard deviation from the mean.
