# Supplementary material for: Immuno-PET imaging of tumor-infiltrating lymphocytes using zirconium-89 radiolabeled anti-CD3 antibody in immune-competent mice bearing syngeneic tumors
Source: PLoS One. 2018 Mar 7;13(3):e0193832. doi: 10.1371/journal.pone.0193832 (PMC5841805; doi:10.1371/journal.pone.0193832)
Supplement: S8 Fig — Representative gating for CD4+ and CD8+ T-cell populations (A) and CD3+ populations (B). Dump channel (B) consists of anti-NK1.1, CD14, and CD19 to gate out NK cells, APCs, and B cells, respectively. Representative (C) and total (D) CD3 median fluorescence intensities for C57BL/6J mice treated with DFO-anti-CD3, unconjugated anti-CD3, or PBS control. (DOCX) [file pone.0193832.s008.docx]

**
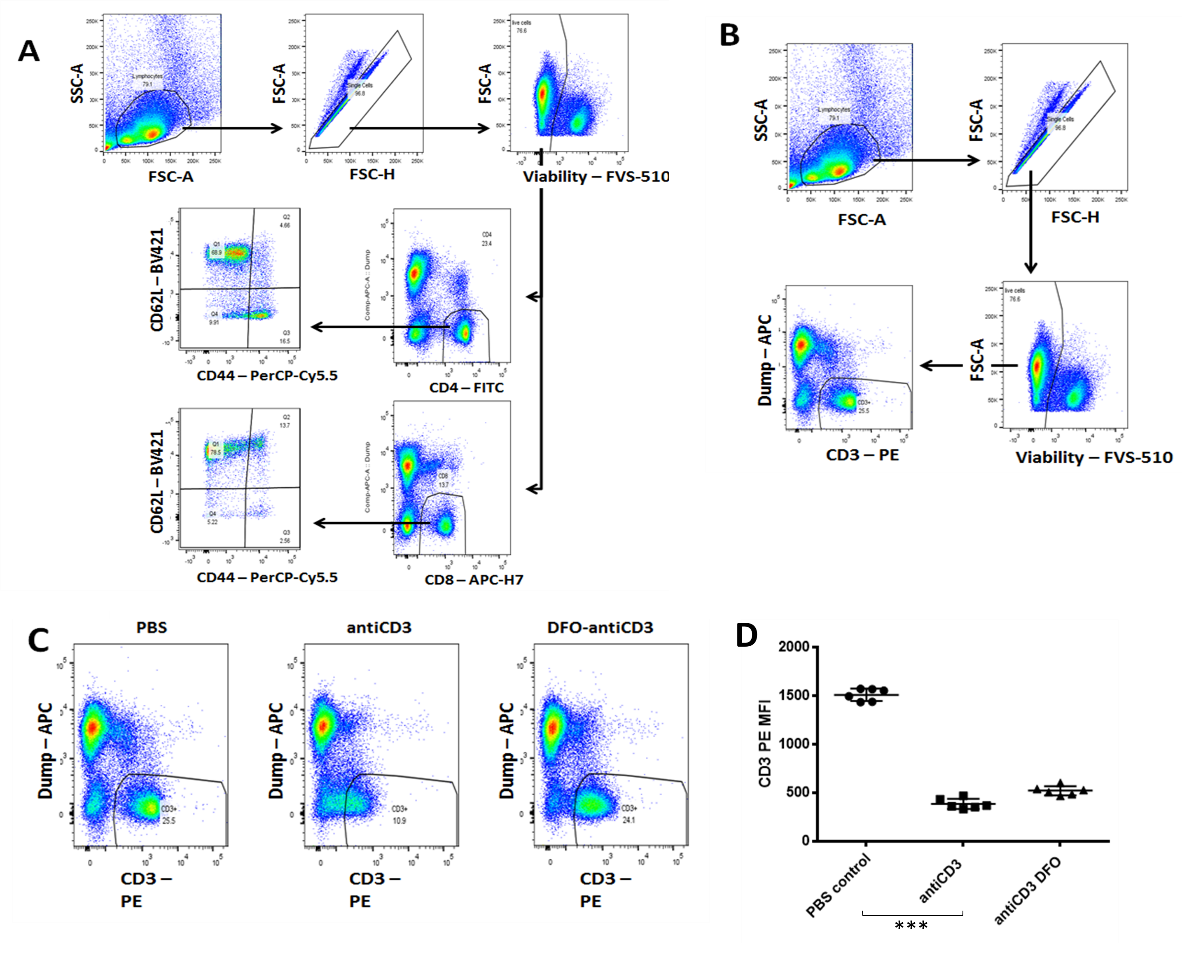
**

**S8 Fig**: **Representative gating for CD4^+^ and CD8^+^ T-cell populations (A) and CD3+ populations (B).** Dump channel (B) consists of anti-NK1.1, CD14, and CD19 to gate out NK cells, APCs, and B cells, respectively. Representative (C) and total (D) CD3 median fluorescence intensities for C57BL/6J mice treated with DFO-anti-CD3, unconjugated anti-CD3, or PBS control.
