# Supplementary material for: Immuno-PET imaging of tumor-infiltrating lymphocytes using zirconium-89 radiolabeled anti-CD3 antibody in immune-competent mice bearing syngeneic tumors
Source: PLoS One. 2018 Mar 7;13(3):e0193832. doi: 10.1371/journal.pone.0193832 (PMC5841805; doi:10.1371/journal.pone.0193832)
Supplement: S9 Fig — Representative gating for CD4+ (top) and CD8+ (bottom) naïve, memory, central memory, and effector memory phenotypes. (DOCX) [file pone.0193832.s009.docx]

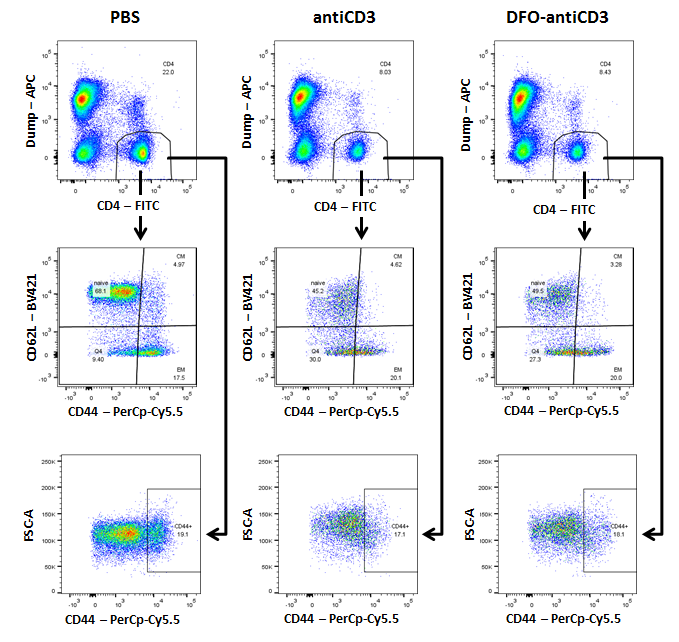

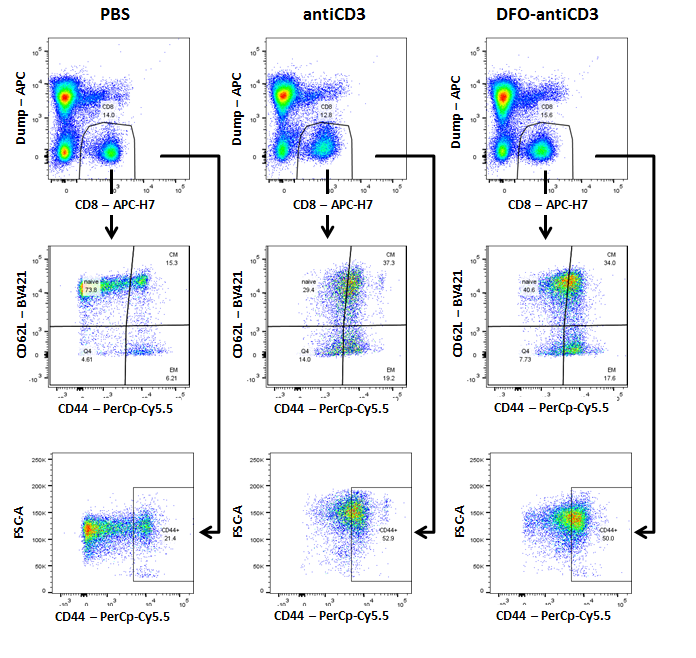


**S9 Fig**: **Representative gating for CD4^+^ (top) and CD8^+^ (bottom) naïve, memory, central memory, and effector memory phenotypes.**
