# Supplementary material for: Immuno-PET imaging of tumor-infiltrating lymphocytes using zirconium-89 radiolabeled anti-CD3 antibody in immune-competent mice bearing syngeneic tumors
Source: PLoS One. 2018 Mar 7;13(3):e0193832. doi: 10.1371/journal.pone.0193832 (PMC5841805; doi:10.1371/journal.pone.0193832)
Supplement: S10 Fig — From left to right: CT, PET and PET-CT with coronal view (top) and transverse view (bottom). Radiolabeled antibody 89Zr-DFO-anti-CD3 was injected in C57BL/6J mice bearing BBN975 tumor and imaged 72h post-injection. T represents the location of the tumor. (DOCX) [file pone.0193832.s010.docx]

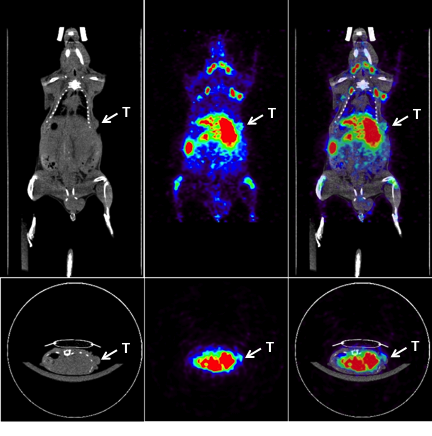


**S10 Fig:** **Second example of tumor infiltrating lymphocytes imaged using micro-PET/CT.** From left to right: CT, PET and PET-CT with coronal view (top) and transverse view (bottom). Radiolabeled antibody ^89^Zr-DFO-anti-CD3 was injected in C57BL/6J mice bearing BBN975 tumor and imaged 72h post-injection. T represents the location of the tumor.
