# Supplementary material for: Immuno-PET imaging of tumor-infiltrating lymphocytes using zirconium-89 radiolabeled anti-CD3 antibody in immune-competent mice bearing syngeneic tumors
Source: PLoS One. 2018 Mar 7;13(3):e0193832. doi: 10.1371/journal.pone.0193832 (PMC5841805; doi:10.1371/journal.pone.0193832)
Supplement: S1 File — Descriptions for reagents and instruments, conjugation and radiolabeling of antibodies, and in vitro serum stability assay. (DOCX) [file pone.0193832.s012.docx]

# Supporting materials and methods

## Reagents and instruments

Anti-CD3 antibody (BE0002) and isotype control Rat IgG 2b (BE0090) were purchased from BioXCell (West Lebanon, NH). Rat IgG (02902) was purchased from Life technologies (Rockford, IL) The bifunctional chelator, p – isothiocyanatobenzyl desferrioxamine (DFO) was purchased from Macrocyclics (Dallas, TX). Zirconium-89 in 1 M oxalic acid was purchased from Washington University (Saint Louis, MO). All other reagents were purchased from Fisher Scientific with the highest purity available. Protein concentration was determined using a Nanodrop 2000/2000c. Radioactivity was measured in a CRC-55tW dose calibrator/well counter (Capintec, NJ) or in an automatic γ-counter (2470, Wizard2, Perkin Elmer, Walthem, MA). Size-exclusion high-performance liquid chromatography (SE-HPLC) was performed at room temperature on a Agilent 1200 series chromatographic system equipped with an online flow γ-ray detector (Lablogic). Samples were injected onto Agilent Bio SEC-5 size exclusion column (5 μm, 300 Å; 7.8 x 300 mm) and Agilent Bio SEC-5 size exclusion column (5 μm, 100 Å; 7.8 x 300 mm) connected in series using EDTA 0.01 M in PBS as the isocratic mobile phase. The flow rate was maintained at 1 mL/min and the elution was monitored by UV spectrophotometer at 254 and 280 nm. Sodium dodecyl sulfate polyacrylamide gel electrophoresis (SDS-PAGE) was carried out in an XCell SureLock^TM^ Mini-Cell electrophoresis system. Matrix Assisted Laser Desorption/Ionization Time of Flight Mass Spectrometry (MALDI-TOF-MS) was performed in a TOF/TOF^TM^ 5800 system using a matrix of Sinapinic Acid. MicroPET/CT experiments were performed in a GE eXplore Vista small animal PET/CT system.

## Conjugation of anti-CD3, IgG and IgG2b with DFO

Modification of anti-CD3, IgG and IgG 2b was done based on literature procedures.^31^ Briefly, the stock solution of anti-CD3 was buffer exchange to 0.1M NaHCO_3_ by ultracentrifugation using a Vivaspin 2 (30kDa molecular weight cut off). Protein concentration was measured using Nanodrop UV spectrophotometry measured at 280 nm. Three milligrams of mAb (2 x 10^5^ mmol, 6 mg/mL) were mixed with 5-fold molar excess of DFO (1 x 10^4^ mmol, 75 μg) previously dissolved in 10 μL of anhydrous DMSO. The reaction mixture was incubated for 45 min at 37 °C with gentle stirring. The conjugates were purified via ultracentrifugation using Vivaspin 2 until the absorbance in the filtrate at 280 nm was nearly zero. Conjugates were characterized by SE-HPLC, SDS-PAGE and MALDI-TOF-MS. Protein concentration was determined by spectrophotometric analysis using bicinchoninic acid assay (BCA). Conjugates were stored at 4°C for further use.

## Radiolabeling of DFO-anti-CD3, DFO-IgG and DFO-IgG2b with ^89^Zr

To 20 μL of water, 15 μL of ^89^Zr [18.5 MBq (0.5 mCi)] and 15 μL of 1M Na_2_CO_3_ were added and allowed to stand for 3 min. Afterward 70 μg (50 μL) of conjugate, previously buffer exchange to 0.5 M HEPES pH-7.4, was added to the reaction mixture and incubated for 1h at RT. Radiolabeled conjugates were purified from unbound radiometal by size exclusion with a PD-10 desalting columns using PBS as the eluent. Fractions of 1mL were collected and radioactivity was measured. Isolated radiolabeling yield was calculated from the ratio of activity in the fractions containing ^89^Zr-DFO-mAb to the initial activity used for radiolabeling. Chemical and radiochemical purity was determined by SE-HPLC and Instant thin layer chromatography (ITLC). ITLC was developed in Sodium Citrate 0.1 M pH-5.5 (Rf = 0 radiolabeled protein and Rf = 1 Free ^89^Zr).

***In vitro serum stability assay***

In vitro stability of ^89^Zr-DFO-CD3 was evaluated in C57BL/6 mouse serum. Briefly, 100 µCi of ^89^Zr-DFO-CD3 in PBS (100 µL) was added to 500 µL of C57BL/6 mouse serum. The sample was incubated at 37^o^C and aliquots were tested for radiochemical purity using iTLC at 1, 24, 48 and 72h. ITLC was developed in Sodium Citrate 0.1 M pH-5.5 (Rf = 0 radiolabeled protein and Rf = 1 Free ^89^Zr). Each experiment was carried out in triplicate.

**
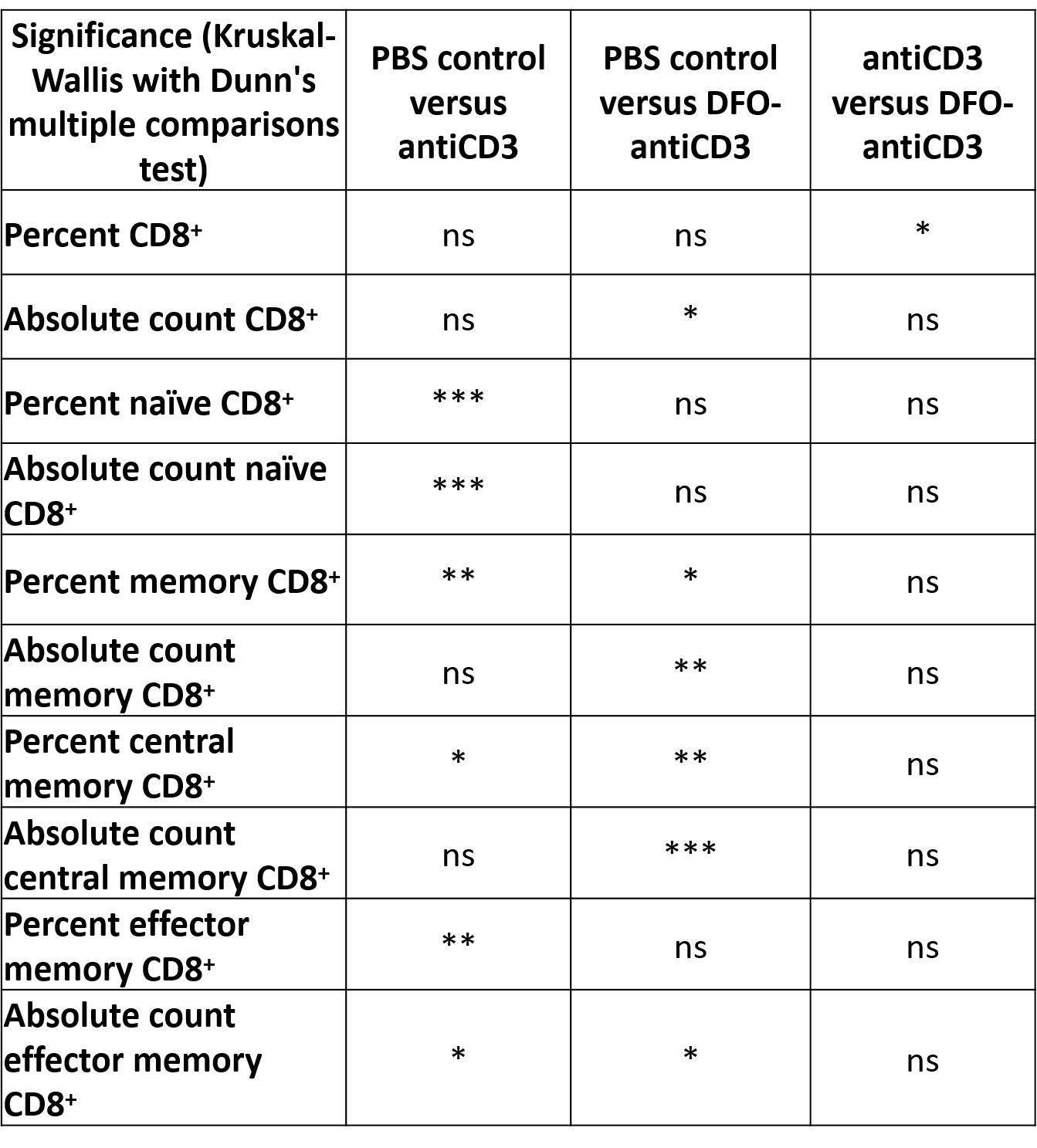
**
