# Supplementary material for: Immuno-PET imaging of tumor-infiltrating lymphocytes using zirconium-89 radiolabeled anti-CD3 antibody in immune-competent mice bearing syngeneic tumors
Source: PLoS One. 2018 Mar 7;13(3):e0193832. doi: 10.1371/journal.pone.0193832 (PMC5841805; doi:10.1371/journal.pone.0193832)
Supplement: S1 Table — Statistical analysis of CD4+ (left) and CD8+ (right) T-cell phenotypes by frequency and absolute count, as determined via Kruskal-Wallis omnibus test with Dunn’s multiple comparisons post-test. Significance is displayed as ns (P > 0.05), * (P ≤ 0.05), ** (P ≤ 0.01), or *** (P ≤ 0.001). (DOCX) [file pone.0193832.s013.docx]

**
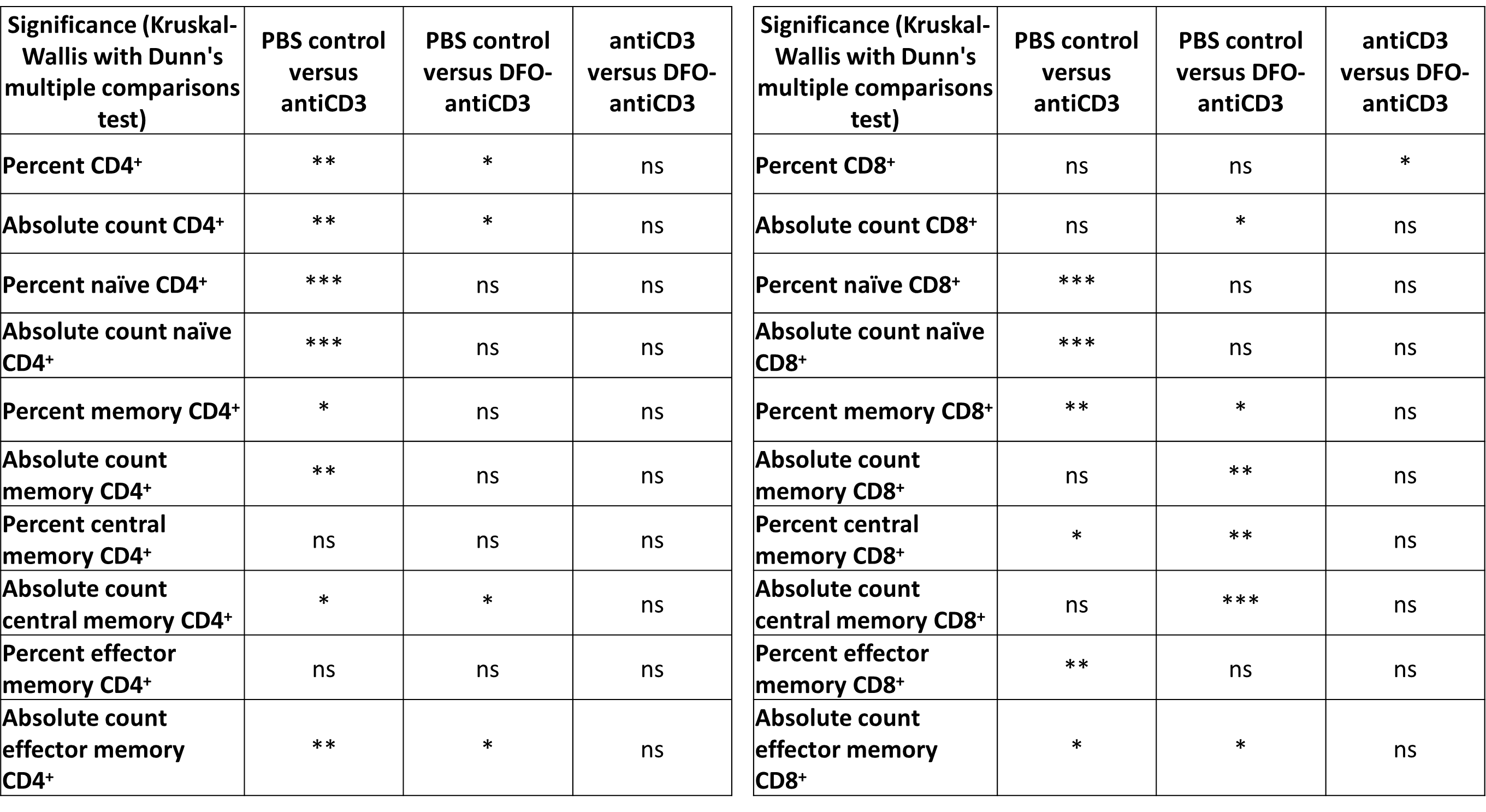
**

**S1 Table: Statistical analysis of CD4^+^ (left) and CD8^+^ (right) T-cell phenotypes by frequency and absolute count, as determined via Kruskal-Wallis omnibus analysis with Dunn’s multiple comparisons post-test.** Significance is displayed as ns (P > 0.05), * (P ≤ 0.05), ** (P ≤ 0.01), or *** (P ≤ 0.001).
